# Lower cholinergic basal forebrain volumes link with cognitive difficulties in schizophrenia

**DOI:** 10.1038/s41386-021-01070-x

**Published:** 2021-06-29

**Authors:** Mihai Avram, Michel J. Grothe, Lena Meinhold, Claudia Leucht, Stefan Leucht, Stefan Borgwardt, Felix Brandl, Christian Sorg

**Affiliations:** 1 Department of Psychiatry and Psychotherapy, Schleswig Holstein University Hospital, University of Lübeck, Lübeck, 23538 Germany; 2 Unidad de Trastornos del Movimiento, Servicio de Neurología y Neurofisiología Clínica, Instituto de Biomedicina de Sevilla, Hospital Universitario Virgen del Rocío/CSIC/Universidad de Sevilla, 41013 Sevilla, Spain; 3 TUM-NIC Neuroimaging Center, Technical University of Munich, School of Medicine, Munich, 81675 Germany; 4 Department of Psychiatry and Psychotherapy, Technical University of Munich, School of Medicine, Munich, 81675 Germany; 5 Department of Neuroradiology, Technical University of Munich, School of Medicine, Munich, 81675 Germany

**Keywords:** Experimental models of disease, Brain

## Abstract

A potential pathophysiological mechanism of cognitive difficulties in schizophrenia is a dysregulated cholinergic system. Particularly, the cholinergic basal forebrain nuclei (BFCN), the source of cortical cholinergic innervation, support multiple cognitive functions, ranging from attention to decision-making. We hypothesized that BFCN structural integrity is altered in schizophrenia and associated with patients’ attentional deficits. We assessed gray matter (GM) integrity of cytoarchitectonically defined BFCN region-of-interest in 72 patients with schizophrenia and 73 healthy controls, matched for age and gender, from the COBRE open-source database, via structural magnetic resonance imaging (MRI)–based volumetry. MRI-derived measures of GM integrity (i.e., volumes) were linked with performance on a symbol coding task (SCT), a paper-pencil-based metric that assesses attention, by correlation and mediation analysis. To assess the replicability of findings, we repeated the analyses in an independent dataset comprising 26 patients with schizophrenia and 24 matched healthy controls. BFCN volumes were lower in patients (*t*(139)=2.51, *p* = 0.01) and significantly associated with impaired SCT performance (*r* = 0.31, *p* = 0.01). Furthermore, lower BFCN volumes mediated the group difference in SCT performance. When including global GM volumes, which were lower in patients, as covariates-of-no-interest, these findings disappeared, indicating that schizophrenia did not have a specific effect on BFCN relative to other regional volume changes. We replicated these findings in the independent cohort, e.g., BFCN volumes were lower in patients and mediated patients’ impaired SCT performance. Results demonstrate lower BFCN volumes in schizophrenia, which link with patients’ attentional deficits. Data suggest that a dysregulated cholinergic system might contribute to cognitive difficulties in schizophrenia via impaired BFCN.

## Introduction

Cognitive difficulties are a major symptom cluster of schizophrenia and include impairments in selective attention, semantic and working memory, and decision-making [1–5]. Among these, attentional deficits have long been considered as a central feature of cognitive difficulties in schizophrenia [6]. The pathophysiological mechanisms underlying cognitive symptoms, including attentional deficits are, however, poorly understood [7, 8]. A potential candidate for such pathophysiological mechanisms is the dysregulation of cerebral cholinergic signaling [9, 10]. The cholinergic basal forebrain nuclei (BFCN) provide the major cholinergic innervation to the prefrontal cortices, the hippocampi, and amygdalae [11, 12]. Its main neurotransmitter is acetylcholine (ACh), which binds to two types of receptors, namely muscarinic (mAChRs) and nicotinic receptors (nAChRs) [12]. Several lines of evidence support the idea that, on the one hand, BFCN integrity and thereby regular cholinergic input is relevant for typical cognitive functioning, and, on the other hand, that cholinergic system alterations might be relevant for attentional deficits in schizophrenia.

There is consistent evidence that BFCN facilitates a wide-range of cognitive processes, ranging from attention to memory, via modulatory effects of ACh on mAChRs and nAChRs [13–15]. Indeed, cholinergic neurons are able to modulate several brain processes via complex, cluster-based, and topographically organized projections into cortical and subcortical regions [11, 16–18]. For example, animal studies have shown that lesioning BFCN projections to the cortex (e.g., via deafferentation) leads to selective impairment of attentional functions [19–21]. Furthermore, performance-related (i.e., transient) increases in ACh have been related to attentional demand as measured by microdialysis in task-performing animals [22, 23]. In line with such findings, it has been put forth that ACh transmission includes both tonic and phasic release, with the former (i.e., volume transmission) being involved in global brain states such as arousal and the latter modulating directly cognitive/behavioral processes such as attention and sensory processing [18, 24]. Whereas tonic ACh release is subject to some debate [24], newer methods such as fluorescent ACh sensor allow for real-time, in vivo monitoring of phasic ACh release—i.e., based on very short timescales (subseconds versus minutes, as required by microdialysis [25, 26])—which is directly linked to behavioral tasks such as cue-reward learning [27]. The timing of ACh release is particularly important for attention, which allows for the detection of relevant cues in the environment and the discrimination of these cues from irrelevant stimuli [28]. This process is thought to rely on ACh enhancement of the signal-to-noise ratio in sensory cortices, thereby increasing neuronal sensitivity to external stimuli (i.e., stimulus-driven attention) [18, 29]. Human evidence is based on approaches employing pharmacological manipulation of the cholinergic system. For example, administration of mAChR antagonists (i.e., scopolamine) was linked to impaired free and cued recall from short and long-term memory in healthy subjects [30, 31]. Correspondingly, administration of nicotine (i.e., nAChR agonist) in healthy subjects undergoing functional magnetic resonance imaging (fMRI) leads to enhanced attention and increased neuronal activity during attentional tasks [32–34]. Indeed, the involvement of ACh signaling in attention is one of the best-documented contributions of the cholinergic system to cognitive processes [29, 35, 36].

In addition to the link between BFCN and normal cognitive processes, there is direct evidence that the cholinergic system is altered in schizophrenia and that its alterations link with cognitive difficulties, including attentional deficits. This evidence is based on two distinct types of findings in schizophrenia: alterations in synaptic cholinergic transmission demonstrated by widespread reductions in mAChRs and nAChRs via neuropathology and molecular imaging, and pharmacological manipulation of the cholinergic system. Regarding the former, postmortem findings have consistently demonstrated that both mAChRs [37–40] and nAChRs [41, 42] are lower in several brain regions linked to cognitive functioning (e.g., prefrontal cortices) in schizophrenia. Although scarce, these findings are supported by in vivo imaging studies using positron emission tomography (PET) or single-photon emission computed tomography (SPECT) also reporting decreased mAChRs [43] and nAChRs [44–46] in patients. Remarkably, lower nAChRs availability in patients was linked to more severe negative symptoms and worse performance on cognitive tests such as Stroop and digit symbol tasks, which assess several cognitive functions, including attention [45, 46]. While similar imaging findings are lacking for mAChRs, improved cognitive function has been reported in patients after administration of xanomeline, a mAChR agonist [47]. Similarly, several pharmaco-fMRI studies have shown that the administration of nicotine to patients increased performance on cognitive tasks assessing working memory and attention [48] and improved accuracy and reaction time during sustained visual attention tasks [49]. In summary, in vivo findings consistently report downregulation of cholinergic receptors in schizophrenia, which might link with patients’ cognitive difficulties, especially attentional deficits. Furthermore, medication, in particular anticholinergic burden (ACB) of pharmacological treatment, has been related to cognitive difficulties in patients with schizophrenia [50], affecting both memory and attention, indicating another link between the cholinergic system and attentional deficits in schizophrenia [51].

Although these studies demonstrate altered synaptic cholinergic transmission in target regions of BFCN projections relevant for attentional deficits, among other cognitive difficulties, it remains unclear, whether the BFCN themselves, as the source of cortical cholinergic innervation, are impaired in schizophrenia. Particularly, it is unclear whether the structural integrity of BFCN is impaired, and whether this might be relevant for patients’ attentional deficits [52–54]. Recent developments in stereotactic mapping using MRI and voxel-based morphometry (VBM) have provided a new way of assessing an in vivo proxy of BFCN structural integrity [55–57]. Applying this method to the field of neurodegenerative and neurodevelopmental disorders has provided evidence for (i) alterations of gray matter (GM) structural integrity of the BFCN (i.e., volume reduction) in Alzheimer’s and Parkinson’s disease [58–61], but also in adults born prematurely [57], and (ii) a link between lower in vivo BFCN volumes and cognitive impairment [57, 60, 62].

Thus, we hypothesized that GM integrity (i.e., volume) of the BFCN is lower in patients with schizophrenia and associated with their attentional deficits. We tested these hypotheses with MRI-based VBM using a stereotactic map of the BFCN [57, 63] in two independent cohorts of patients with schizophrenia and healthy controls, and examined the association between GM alterations and attentional deficits measured by the symbol coding task (SCT) of the Brief Assessment of Cognition in Schizophrenia, a paper-pencil-based metric that evaluates attention and processing speed [64], via correlation and mediation analysis. SCT is highly sensitive for attention deficits in schizophrenia [65–67], which are an excellent predictor of real-world outcomes [10, 68].

## Materials and methods

### Participants

Imaging and clinical-behavioral data of patients with schizophrenia and healthy controls were obtained from the open-source Center for Biomedical Research Excellence (COBRE) database (http://fcon_1000.projects.nitrc.org/indi/retro/cobre.html). 72 patients with schizophrenia receiving antipsychotic medication (Table S1) and meeting DSM-IV criteria (age range: 18–65 years; mean: 38.16 ± 13.89 years) along with 73 controls (age range: 18–65 years; mean: 35.60 ± 11.50 years) were included (Table 1). Diagnosis of schizophrenia was determined with the Structured Clinical Interview for DSM-IV [69]. Controls had no personal history of Axis I disorders, substance abuse, nor first-degree relatives with a history of psychosis.

**Table 1:** Participant demographics and clinical-neuropsychological scores.

	COBRE cohort		*P*-value	Munich cohort		*P*-value
	SCZ	HC		SCZ	HC	
	Mean ± SD			Mean ± SD		
*N*	72	73		26	24	
Age [years]	38.16 ± 13.89	35.60 ± 11.50	0.82	42.84 ± 11.38	38.54 ± 11.63	0.19
Females/males	14/58	23/50	0.55	8/18	9/15	0.61
Illness duration [years]	15.83 ± 12.45		N/A	15.38 ± 9.32		N/A
Chlorpromazine equivalents [mg]	371.5 ± 311.68		N/A	513.96 ± 401.65		N/A
SCT [a.u.]	43.94 ± 11.66	62.18 ± 9.86	<0.001*	42.07 ± 12.25	61.37 ± 12.95	<0.001*
PANSS [a.u.]						
Positive	14.95 ± 4.82		N/A	10.65 ± 3.22		N/A
Negative	14.52 ± 4.82		N/A	14.50 ± 6.01		N/A
BFCN mm^3^	286.80 ± 32.15 (*N* = 69)	298.91 ± 24.75 (*N* = 72)	0.01*	289.84 ± 23.41 (*N* = 26)	303.34 ± 22.98 (*N* = 24)	0.04*

Age and SCT scores were compared with two-sample *t*-tests; sex via chi-squared test.

*SCZ* patients with schizophrenia, *HC* healthy controls, *SCT* symbol coding task, *SD* standard deviation, *N* number of participants, *N/A* not applicable, *PANSS* Positive and Negative Syndrome Scale, *BFCN mm*^3^ intracranial-normalized basal forebrain cholinergic nuclei volumes.

*Statistical significance at *p* < 0.05.

Data of the replication sample was acquired by our group in Munich. Briefly, 26 patients with schizophrenia receiving antipsychotic medication (Table S2) and meeting DSM-IV criteria (age range: 23–65 years; mean: 42.84 ± 11.38 years) and 24 healthy controls (age range: 25–62 years; mean: 38.54 ± 11.63 years), comparable regarding age and sex with the patient sample, were included. Diagnosis of schizophrenia was determined with the Structured Clinical Interview for DSM-IV [69]. Patients were in symptomatic remission of psychotic symptoms [70]. The study was approved by the Ethics Review Board of Klinikum rechts der Isar of the Technical University Munich, and subjects completed their written informed consent.

### Cognitive assessment

Participants’ cognitive abilities were evaluated with the SCT of the Brief Assessment of Cognition in Schizophrenia, which measures attention and speed of processing [64]. In addition to the SCT, participants were also evaluated with the Trail Making Test Part A (TMT-A), which provides information on visual search and speed of processing [71]. We used TMT-A as control, to disentangle whether patients’ attentional deficits or rather their deficits in the speed of processing link with putative changes in BFCN volumes.

### MRI data processing

**Fig. 1:**
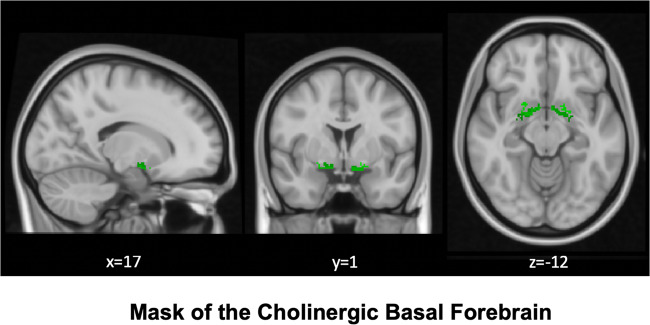
Map of the cholinergic basal forebrain. The region of interest depicts the cholinergic basal forebrain, based on a cytoarchitectonic map of cholinergic nuclei, overlaid on a human brain template in Montreal Neurological Institute space. The BFCN mask is based on combined histology and postmortem MRI [63], containing several cholinergic subdivisions within the basal forebrain, including the medial septal nucleus, diagonal band of Broca, nucleus subputaminalis, the basal magnocellular complex, and nucleus basalis of Meynert [57, 72].

Structural MRI data were processed using the CAT12 toolbox (http://dbm.neuro.uni-jena.de/cat/) implemented in SPM12 (http://www.fil.ion.ucl.ac.uk/spm/software/spm12/). We used the pre-set, default parameters for preprocessing, in line with the standard protocol (http://www.neuro.uni-jena.de/cat12/CAT12), unless otherwise specified. Briefly, images were first segmented into GM, white matter (WM), and cerebrospinal fluid (CSF) partitions of 1.5 mm isotropic voxel size. Each participant’s GM and WM and CSF images were then registered to stereotactic standard space (Montreal Neurological Institute) and then smoothed with a Gaussian kernel full width half maximum (FWHM) of 8 mm. Preprocessed GM maps were visually inspected for segmentation and registration accuracy. Total intracranial volume (TIV) calculated as the sum of the total GM, WM, and CSF volumes was used to control for differences in head size in all analyses. Next, individual GM maps reflecting volumes were extracted from warped GM segments by averaging voxels within an a priori BFCN region-of-interest (ROI) (Figs. 1 and S1). The BFCN ROI is based on a stereotactic atlas of basal forebrain cholinergic nuclei derived from cytoarchitectonic mappings, based on combined histology and postmortem MRI [63], containing several cholinergic subdivisions within the basal forebrain, including the medial septal nucleus, diagonal band of Broca, nucleus subputaminalis, the basal magnocellular complex, and nucleus basalis of Meynert [57, 72].

### Statistical analyses

#### Group differences in BFCN volumes

Statistical analyses were performed with SPSS version 21 (IBM Corp., Armonk, NY). Group differences between healthy controls and patients with schizophrenia in intracranial volume-normalized BFCN, intracranial volume-normalized GM, intracranial volume-normalized WM, and SCT scores were evaluated with two-sample *t*-tests (*p* < 0.05, two-sided). The TIV-scaled, intracranial volume-normalized BFCN, global GM, and global WM were used in all subsequent analyses. Participants with extreme values in BFCN volumes (>1.5× interquartile range) were excluded from further analyses (*N* = 4, see below). Group differences in sex were evaluated with the chi-squared test. Analysis of covariance (ANCOVA) was used to assess the (relative) specificity of group differences in BFCN volumes, controlling for the influence of global GM and WM volumes, age, smoking, and ventricle size, respectively. Spearman’s correlation analysis was used to investigate the associations between BFCN volumes and chlorpromazine equivalents (CPZ) in patients. Pearson correlation analysis was used to investigate associations between BFCN volumes and psychotic symptoms (as measured by PANSS) in patients.

#### Associations between BFCN volumes and SCT in patients

(i) Patients that had missing SCT scores (*n* = 4) were excluded from the analyses evaluating associations between BFCN and attentional deficits. Shapiro-Wilk was used to determine whether data were normally distributed. Pearson correlation was used to asses associations between BFCN volumes and SCT scores in patients. We used partial correlations to control for possible influences of global GM and WM volumes, ACB of medication, and smoking on the association between BFCN volumes and SCT scores. Smoking was included as a dummy variable (0 for non-smoker and 1 for smoker) in these analyses.

(ii) To test whether the attentional deficits seen in patients were mediated by lower BFCN volumes, we performed mediation analysis with the PROCESS package [73]. For detailed description see Supplementary Methods. To control for possible influences of global GM volumes and smoking on the relationship between BFCN volumes and group difference in SCT, we repeated the analysis and added global GM and smoking, respectively, as a covariates-of-no-interest in the models.

### Control and specificity analyses

To ensure that putative differences in BFCN volumes and their link to patients’ attentional deficits were not driven by some methodological artefact, and to better understand our results in terms of relative specificity, we performed several analyses for the following factors: the effect of ROI-based versus voxel-based approaches, regional specificity for BFCN sub-regions, the influence of anticholinergic medication effects, the specificity of cognitive assessment, the specificity of global GM influence on regional volume changes, and the replicability of findings in other patient samples. See Supplementary Methods for details.

## Results

### Cholinergic basal forebrain volumes are lower in patients with schizophrenia

**Fig. 2:**
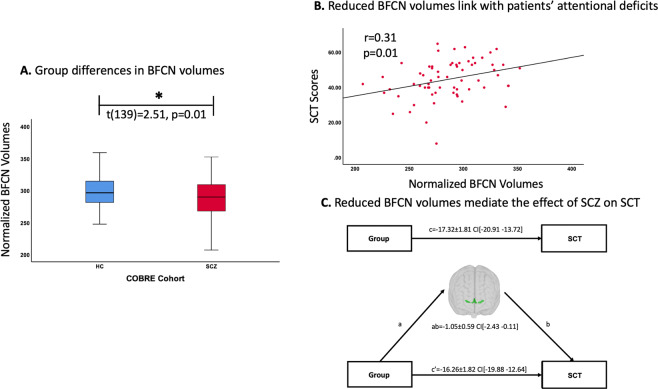
Lower BFCN volumes in patients with schizophrenia are associated with attentional deficits in the COBRE cohort. **A** Group differences in BFCN volumes. Group comparisons between healthy controls (blue box) and patients with schizophrenia (red box) regarding BFCN volumes were computed with a two-sample *t*-test on the intracranial-normalized BFCN volumes with *p* < 0.05 (two-tailed). Groups differed significantly in BFCN volumes (*t*(139)=2.51, *p* = 0.01). **B** Associations between lower BFCN volumes and patients’ attentional deficits. Associations between BFCN volumes and SCT scores were computed with Pearson correlation analysis with *p* < 0.05 (two-tailed). Plot depicts a significant correlation between BFCN values and SCT scores (*r* = 0.31, *p* = 0.01). **C** Lower BFCN volumes mediate the group difference in SCT. BFCN volumes mediate the association between group (patients versus healthy controls) and SCT scores. Two models are depicted by the path diagrams: path c, which indicates the group difference in SCT scores (top), compared to path ab, which indicates that lower BFCN volumes mediate the effect of group on SCT (bottom). The regression coefficients (unstandardized) for each effect are shown (±standard error). Significance is depicted with confidence intervals (CI). BFCN cholinergic basal forebrain nuclei, ROI region-of-interest, SCT symbol coding task, HC healthy controls, SCZ patients with schizophrenia.

In the COBRE cohort, four participants (one control and three patients) were excluded from further analyses, due to extreme values (>1.5× interquartile range) in BFCN volumes. The remaining participants, 72 controls and 69 patients did not differ in age as demonstrated by a two-sample *t*-test (*t*(139)=−0.97, *p* = 0.33), and only at-trend in sex as shown by a chi-squared test (*X*^2^(1,141)=3.18, *p* = 0.07). Patients had lower BFCN volumes (−3.4%) compared to controls. This difference was statistically significant as demonstrated by a two-sample *t*-test on BFCN volumes (*t*(139)=2.51, *p* = 0.01; Fig. 2A).

Next, we tested whether the lower BFCN volumes observed in patients were potentially influenced by other variables. First, we tested for differences in global GM and WM. Two-sample *t*-tests demonstrated that patients differed from controls in global GM (*t*(139)=3.20, *p* = 0.002)—i.e., had lower volumes, but not in global WM (*t*(139)=0.19, *p* = 0.84). To test whether global WM or GM might influence the group difference in BFCN volumes, we computed ANCOVAs with global WM and GM, respectively, as covariates-of-no-interest. The group differences in BFCN volumes remained significant when controlling for global WM (*F*_1,138_ = 6.34, *p* = 0.01), indicating that global WM did not influence the group difference. The group differences in BFCN volumes were no longer significant after controlling for global GM (*F*_1,138_ = 0.82, *p* = 0.35), indicating an effect of lower global GM volumes on the group difference in BFCN volumes. To better understand this global GM influence, we tested whether global GM also influences volume changes of other brain regions in patients. Specifically, using the same approach as for BFCN volumes, we tested whether global GM affects the group difference in anterior cingulate cortex (ACC), insula, thalamus, and striatum, respectively (see Supplementary Results for details). Crucially, we found that all ROIs except of the striatum had lower volumes in patients, but that the group differences were no longer significant when controlling for global GM. These results indicate that global GM does not only contribute to lower BFCN volumes but also to those of other regions such as ACC or insula. We conclude that in patients with schizophrenia lower global GM volumes affect multiple GM regions, including BFCN volumes.

Next, ANCOVAs using sex, age, and smoking, respectively, instead of global GM/WM as covariate-of-no-interest demonstrated that these variables did not influence the group differences in BFCN volumes (see Supplemental Results). However, when controlling for ventricle size (ventricles were enlarged in patients), the group difference in BFCN volumes was no longer significant, indicating that ventricular enlargement influences the group difference in BFCN volumes.

Current antipsychotic medication (CPZ) was not associated with lower BFCN volumes, as demonstrated by Spearman’s correlation analysis (rho = −0.20, *p* = 0.09), suggesting that antipsychotic medication did not influence the group difference in BFCN volumes. Furthermore, no associations were found between lower BFCN volumes and psychotic symptoms (as measured by PANSS) with Pearson correlation analyses (*r* = −0.11, *p* = 0.36), suggesting that the group difference in BFCN volumes was not driven by psychotic symptoms.

Finally, in order to specify the regional influence on patients’ lower BFCN volumes, we performed voxel-wise analysis to locate the peak differences within the BFCN ROI. Results demonstrated lower BFCN volumes in patients, peaking in the medial septal nucleus. This finding was supported by a control analysis, which investigated anterior and posterior clusters of an alternative BFCN ROI mask (see Supplemental Results and Fig. S2).

### Lower cholinergic basal forebrain volumes are associated with attentional deficits in patients

Next, we assessed whether lower BFCN volumes were associated with attention, namely performance on SCT, in patients. SCT were significantly reduced in patients (*t*(128) = 9.53, *p* < 0.001), indicating impaired attention. A Shapiro-Wilk test demonstrated that SCT scores were normally distributed in the patient group (*p* = 0.1). Pearson correlation between BFCN volumes and SCT scores revealed a significant association between lower BFCN volumes and impaired SCT performance (*r* = 0.31, *p* = 0.01; Fig. 2B). This result suggests that lower BFCN volumes are relevant for patients’ attentional deficits. In addition, both anterior and posterior BFCN volumes were associated with SCT scores in patients, with a slightly stronger effect for the posterior BFCN (see Supplementary Results).

Next, we tested whether global GM and WM influence the relation between lower BFCN and attentional deficits. Initially, we investigated whether global GM and WM, respectively, were associated with SCT scores in patients, using Pearson correlation analysis. SCT scores correlated with global GM (*r* = 0.45, *p* < 0.001), but not with global WM (*r* = 0.21, *p* = 0.09). To test whether the association between BFCN volumes and SCT scores was influenced by global GM and WM, we computed a partial correlation analysis between BFCN volumes and SCT scores, controlling for global GM and WM, respectively. Concerning global WM, the association between BFCN volumes and SCT scores remained significant (*r* = 0.32, *p* = 0.008), indicating that the association was independent of global WM. The relationship was no longer significant after controlling for global GM (*r* = 0.09, *p* = 0.45), indicating that the correlation between BFCN volumes and SCT scores in patients was influenced by global GM. To better understand this influence of global GM, we investigated whether global GM also influences the relationship between lower volumes of other regions and SCT scores. We found that the lower volumes of ACC and insula were linked with patients’ SCT scores, respectively, indicating that these volume changes are relevant for patients’ attentional deficits (see Supplementary Results). We did not find a similar link for the lower thalamus volume, indicating that not all regions with lower volumes are relevant for attentional deficits. Remarkably, using the same partial correlation approach as for the BFCN, correlations between ACC/insula volumes and SCT scores were no longer significant in patients, when controlling for global GM, indicating that lower global GM volumes influence the association between SCT scores and lower BFCN, ACC, and insula volumes, respectively. These results suggest that several regions that have lower volumes in patients are relevant for the impaired SCT performance, similar to lower global GM.

Next, we controlled for the putative effect of ACB of medication on the association between BFCN volumes and SCT. A Shapiro-Wilk test demonstrated that ACB scores (Table S1) were not normally distributed (*p* = 0.001). We therefore computed a Spearman partial correlation, to investigate the effect of ACB on the relationship between BFCN volumes and SCT scores. The association between BFCN volumes and SCT scores remained significant (rho = 0.23, *p* = 0.008), indicating that the association is independent of ACB.

We then tested whether smoking might have an effect on the relationship between lower BFCN volumes and lower SCT scores in patients. A Shapiro-Wilk test demonstrated that smoking was not normally distributed (*p* = 0.001). We therefore computed a Spearman partial correlation which demonstrated that the association between BFCN volumes and SCT scores remained significant (rho = 0.33, *p* = 0.007) in patients.

Next, we investigated whether BFCN volumes were also related to SCT scores in healthy controls. A Shapiro-Wilk test demonstrated that SCT scores were normally distributed in the control group (*p* = 0.28). Pearson correlation analysis demonstrated that the association between BFCN volumes and SCT scores was not significant in controls (*r* = 0.08, *p* = 0.50), suggesting a schizophrenia-specific link between lower BFCN volumes and SCT performance.

Finally, we evaluated whether BFCN volumes link specifically with attention rather than speed of processing, by investigating the relationship between BFCN volumes and TMT-A scores in patients. The relationship between lower BFCN volumes and TMT-A scores was not significant (see Supplementary Results), indicating a specific link between lower BFCN volumes and attentional deficits, as measured by SCT.

#### Mediation analysis

To further analyze the link between BFCN volume alterations and attention deficits in patients, we tested whether lower BFCN volumes mediate the group difference in SCT via mediation analysis. Mediation analysis revealed a significant indirect effect of group on SCT via BFCN volumes (ab = −1.05 ± 0.59; the bootstrapped 95% confidence interval CI[−2.43 −0.11]; Fig. 2C), indicating a significant mediation of the group difference in SCT via lower BFCN volumes. We conclude that lower BFCN volumes likely contribute to attentional deficits in schizophrenia. Furthermore, we found a significant effect both for the anterior and posterior BFCN clusters, indicating that the whole BFCN contributes to attentional deficits (see Supplementary Results). We tested whether BFCN volume mediation of the group difference in SCT scores was affected by smoking by including smoking as a covariate-of-no-interest in the model. The indirect effect remained significant (ab = −0.78 ± 0.50; the bootstrapped 95% CI[−0.94 −0.02]), indicating that the mediation is independent of smoking status. To control for the putative effect of lower global GM volumes, we repeated the mediation analysis but included global GM as a covariate-of-no-interest in the model. The indirect effect was no longer significant (ab = −0.01 ± 0.24; the bootstrapped 95% CI[−0.65 0.38]), indicating that lower global GM influences the relationship between BFCN volumes and group difference in SCT. As before, to better understand the influence of global GM on the mediation effect of BFCN volumes, we repeated the mediation analyses for ACC and insula volumes, respectively (see Supplementary Results). We found that ACC and insula volumes mediated the group difference in SCT scores, respectively, but these mediations were no longer significant when global GM was added as covariate-of-no-interest in the models. These results suggest that while both ACC and insula volumes are related to attentional processes, global GM modulates these relationships. Indeed, we found that global GM also mediated the group difference in SCT scores, but that this mediation was no longer significant after controlling for BFCN, ACC, and insula volumes, respectively. These findings indicate a complex relationship between global GM and ROIs relevant for attentional deficits, suggesting that there is not one region that drives the association with attentional deficits, but rather that several brain regions might contribute to such an effect.

### Replication study in the Munich cohort

**Fig. 3:**
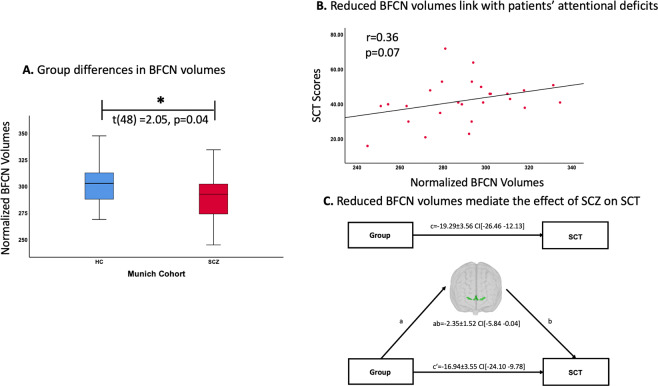
Lower BFCN volumes in patients with schizophrenia are associated with attentional deficits in the Munich cohort. **A** Group differences in BFCN volume. Group comparisons between healthy controls (blue box) and patients with schizophrenia (red box) regarding BFCN volumes were computed with a two-sample *t*-test on the intracranial-normalized BFCN volumes with *p* < 0.05 (two-tailed). Groups differed significantly in BFCN volumes (*t*(48) = 2.05, *p* = 0.04). **B** Associations between lower BFCN volumes and patients’ attentional deficits. Associations between BFCN volumes and SCT scores were computed with Pearson correlation analysis with *p* < 0.05 (two-tailed). Plot depicts an at-trend-to-significant association between BFCN volume values and SCT scores (*r* = 0.36, *p* = 0.07). **C** Lower BFCN volumes mediate the group difference in SCT. BFCN volumes mediate the association between group (patients versus healthy controls) and SCT scores. Two models are depicted by the path diagrams: path c, which indicates the group difference in SCT scores (top), compared to path ab, which indicates that lower BFCN volumes mediate the effect of group on SCT (bottom). The regression coefficients (unstandardized) for each effect are shown (±standard error). Significance is depicted with confidence intervals (CI)*.* BFCN cholinergic basal forebrain nuclei, ROI region-of-interest, SCT symbol coding task, HC healthy controls, SCZ patients with schizophrenia.

To assess the replicability of our findings, we used the identical approach in the Munich cohort. We found that BFCN volumes were lower in patients (−4.3%) compared to controls. This difference was statistically significant as demonstrated by a two-sample *t*-test on BFCN volumes (*t*(48) = 2.05, *p* = 0.04; Fig. 3A). Next, Pearson correlation analysis was used to investigate the relationship between lower BFCN volumes and SCT scores in patients. The correlation between BFCN volumes and SCT scores was at-trend-to-significant in patients (*r* = 0.36, *p* = 0.07; Fig. 3B). This relationship was not significant in healthy controls (*r* = 0.30, *p* = 0.14). Finally, mediation analyses demonstrated that lower BFCN volumes mediated the effect of schizophrenia on SCT performance (ab = −2.35 ± 1.50; the bootstrapped 95% CI[−5.84 −0.04]; Fig. 3C). Akin to the COBRE findings, we found that controlling for global GM volumes had an effect on all main results. In summary, we replicated our main findings in an independent sample of patients and controls, supporting the reliability of our results. For detailed description of the replication analyses see Supplementary Results.

## Discussion

Using MRI-based volumetry together with a cytoarchitectonically defined mask of the BFCN, we provide first-time evidence for lower structural GM integrity of the BFCN in patients with schizophrenia, and show that the lower volumes contribute to patients’ attentional deficits. Our findings suggest that a dysregulated cholinergic system might contribute to cognitive difficulties in schizophrenia via impaired BFCN.

### Lower BFCN volumes in schizophrenia

Our first major finding was that BFCN volumes were lower in patients with schizophrenia (Fig. 2A). Crucially, this result was robust and reproducible in an independent dataset (Fig. 3A). In addition, we found that BFCN volumes were consistently lower both in the anterior and posterior BFCN clusters, with more accentuated group differences in the anterior BFCN. In line with this finding, voxel-wise analysis revealed lower volumes across BFCN regions with more prominent changes in the medial septal nucleus (Fig. S2). These findings indicate widespread volume reduction across the whole BFCN. Furthermore, lower BFCN volumes in patients were not influenced by age, ACB of medication, smoking, and antipsychotic medication. We also found that lower BFCN volumes were independent of psychotic episodes (in the Munich cohort patients were in remission of psychosis) and severity of psychotic symptoms. These findings indicate that lower BFCN volumes reflect rather a trait of schizophrenia than a state of psychosis.

Although lower BFCN volumes were not influenced by changes in global WM, there was a significant effect of global GM on the group difference in BFCN volumes. Remarkably, we found similar results for alternative regions that had lower volumes, such as the ACC and insula cortices—i.e., when controlling for global GM group differences in ACC and insula volumes were no longer significant. This suggests that multiple regions that have lower volumes in schizophrenia, including the BFCN, are not independent of the degree of averaged volume loss across the whole brain. The finding of lower BFCN volumes adds to the growing literature providing consistent evidence for regionally selective lower GM volumes in patients with schizophrenia, including subcortical structures such as amygdala and thalamus [74–77]. One should note that increases in GM have also been reported, mainly in the basal ganglia and in association with psychosis and antipsychotic medication [75, 78–80]. In line with these findings, our specificity analyses revealed lower volumes in the ACC, insula, and thalamus, but not striatum [76, 77]. Furthermore, the result of lower BFCN volumes in patients with schizophrenia is well in line with and extends previous postmortem findings and PET and SPECT studies reporting lower nicotinic and muscarinic receptor availability in patients with schizophrenia [37–46]. Therefore, our finding of lower BFCN volumes supports the hypothesis of an impaired cholinergic system in schizophrenia.

### Lower BFCN volumes are related with attentional deficits in schizophrenia

Our second major finding was that lower BFCN volumes were associated with patients’ impaired performance on SCT in both patients with psychosis and patients in remission of psychosis (Figs. 2B and 3B). This is crucial, as cognitive difficulties, including attentional deficits, are largely not influenced by antipsychotic treatment and still present in remission [7]. Regarding BFCN sub-regions, we found that the association with attentional scores were present both for anterior and posterior BFCN and therefore not specific for a certain cluster. Furthermore, we found that the link between lower BFCN volumes and SCT scores, reflects an association between impaired BFCN and attentional deficits rather than impairment in processing speed, as lower BFCN volumes did not correlate with TMT-A scores, which mainly reflect the speed of processing [71].

Control analyses demonstrated that the link between lower BFCN volumes and patients’ attentional deficits was not influenced by global WM, but global GM changes did affect this association, in a similar way as they influenced the association between impaired SCT performance and lower ACC and insula volumes. This finding suggests that BFCN volumes are relevant for patients’ attentional functions, along with other regions, but also that the relevance for attention is not independent of lower global GM. Of note, the relationship between lower BFCN volumes and attentional deficits in patients remained significant even after controlling for the ACB of all medications (i.e., ACB scores), indicating that lower BFCN volumes link with patients’ attentional deficits independent of medication effects. Furthermore, a mediation analysis demonstrated that the effect of schizophrenia on SCT performance was mediated via lower BFCN volumes (Fig. 2C). The mediation was replicated in the independent Munich dataset (Fig. 3C), indicating that lower BFCN volumes contribute to attentional deficits in schizophrenia. As for the previous analyses, we found that global GM changes influenced the mediation of the group difference in SCT scores via lower BFCN volumes. This was also the case for the ACC and insula volumes. Furthermore, we found that global GM volumes also mediated the group difference in SCT scores. This was not surprising, considering that lower global GM volume, along with a relationship between lower GM volume and cognitive deficits, is a typical finding in psychotic disorders [74, 75, 81, 82]. However, these results are not contrary to our hypothesis that BFCN volumes contribute to attentional deficits in schizophrenia. Indeed, as we have shown, the mediation of global GM volumes on the group difference in SCT scores was no longer significant after controlling for BFCN volumes, demonstrating BFCN involvement.

The contribution of BFCN volumes to cognitive tasks (i.e. attentional performance) is supported by previous reports demonstrating that lower nAChRs availability in patients linked to worse performance on cognitive tests, which also measured attention [46], but also with other imaging findings demonstrating that administration of nicotine to patients increased performance on cognitive tasks assessing working memory and attentional control [48] and improved accuracy and reaction time during sustained visual attention tasks [49]. Interestingly, although available evidence from in vivo studies suggests that nAChRs alterations link with cognitive difficulties, there is also evidence that the muscarinic subsystem is involved in distinct cognitive processes, and that cognitive performance in patients might improve after administration of an mAChR agonist (i.e., xanomeline) [47]; remarkably, however, xamoneline did not improve attentional performance. Taken together, these findings indicate a stronger involvement of nAChRs in attention, however, the findings presented in this study do not allow for a more in-depth interpretation of which ACh receptor system might modulate attentional processes, since the outcome measure used here lacks such sensitivity.

Interestingly, SCT scores were not associated with BFCN volumes in healthy controls in neither cohort, suggesting a schizophrenia-specific link between impaired BFCN volume and impaired attention, which is supported by the mediation analysis. We can only speculate why this relationship was not significant in healthy controls. It is possible that the association seen in patients reflects rather a relationship between altered cholinergic neuron number/ decreased cholinergic projections and diminished attentional capacity than a relationship between cholinergic neuron function and attention per se. Indeed, both BFCN volumes and SCT scores had increased variance compared to healthy controls, and lower minimum values (Table S3 and Fig. S4). Furthermore, small volumetric differences among controls might only weakly indicate a contribution to cognitive functioning. Put differently, in the absence of a pathologic condition, volume does not necessarily equal function. Rather, a pathologic condition, e.g., altered neurodevelopment or degeneration of the BFCN, might lead to related volumetric and cognitive differences that are revealed by the association. In support, a previous study reported a significant relationship between lower BFCN volumes and lower IQ in preterm-born subjects, but no corresponding association was found in term-born individuals [57]. On a similar note, while variance in hippocampal volumes has been linked with memory performance in several neurologic conditions [83, 84], such associations appear to be lacking in healthy subjects [85]. Finally, the association seen in patients might be amplified by the involvement of other attentional structures that present with lower volumes in patients (i.e., ACC, insula) [76, 77]. In support, the cholinergic system’s effect on attention is exerted via several brain regions including cingulate-insular cortices [86–88], which were also found to be altered in this study.

In summary, we showed that BFCN volumes are lower in chronic schizophrenia and linked with patients’ attentional deficits. The neurobiological causes of this reduction remain to be determined. For instance, there is evidence that GM reductions worsen with illness duration, which might suggest an effect of illness and/or long-term treatment with antipsychotic medication [75, 89]. On the other hand, there is also evidence that BFCN volume reduction is linked with impairments in normal development. For example, an abnormal development of the BFCN has been put forth as a vulnerability factor for psychiatric disorders, including schizophrenia [90]. It therefore remains unclear whether BFCN volume reductions reflect an aberrant neurodevelopmental process and/or rather a ‘chronic-neurodegenerative’ one. Future studies need to investigate subjects at clinical high-risk of psychosis and medication-naive first-episode patients and test whether the reduction is already present before onset of psychosis or treatment with antipsychotic medication or if it varies as a function of illness duration.

### Limitations

BFCN measurements of GM volume only indirectly assess the cholinergic system’s integrity, as they rely on stereotactic maps of the BFCN [63]. Therefore, we cannot exclude that lower BFCN volumes were not related to alterations in other neuronal populations within the ROI (i.e., glutamatergic and GABAergic neurons), with which cholinergic neurons mix and interact [91].

Next, structural MRI is not a direct measure of brain anatomy, and is susceptible to several factors that have been reported to alter MRI signals [92, 93].

Furthermore, and as reported above, we found that controlling for global GM had an effect on all main outcomes investigated in this study, in both cohorts (see Supplementary Results), indicating a twofold confounding effect: (a) lower global GM co-varies with the regional differences found for the “attentional ROIs” and (b) lower global GM co-varies with the group difference in attentional performance.

These limitations indicate that it is not the BFCN in particular which are smaller in schizophrenia, but rather that lower global GM co-varies with this effect and is associated with attentional deficits in patients. Regarding (a) volume differences, while we consider it somewhat unlikely that all regional differences found for the ROIs investigated in this study are driven solely by lower overall GM, and that the ROIs themselves are unaffected, future studies are needed to clarify this issue. Perhaps investigating patients that do not differ from healthy controls in global GM will clarify this aspect. Regarding (b) the relevance for attentional deficits, our results demonstrate a widespread effect of global GM on all investigated “attentional ROIs”. Indeed, a whole-brain multiple regression analysis, which investigated the relationship between every GM voxel and SCT performance supports the results of the ROI-based findings (see Supplementary Discussion and Fig. S5). Future studies should employ methods better suited to disentangle global GM from BFCN effects, such as multivariate approaches in which the participants’ GM is parcellated into several regions and the relationship of these volumes is investigated simultaneously with one or more attentional scores—for instance via multivariate pattern analysis [94]. Alternatively, resting-state functional connectivity might be employed to investigate BFCN alterations and their relevance for attention in schizophrenia, as functional connectivity is relatively independent of GM volume. For more details on disentangling the effect of global GM from that of BFCN see supplementary Discussion. However, the main issue of the relative-specificity of BFCN findings, relative to GM volume changes in patients, begs the question, whether we can expect specific BFCN changes in schizophrenia. Although the cholinergic system is affected in schizophrenia, there is little evidence to indicate that schizophrenia specifically targets the BFCN. Furthermore, there is little reason to expect that cholinergic deficits (let alone the BFCN volume as a rough structural proxy for these deficits) are the sole contributors to attentional deficits in schizophrenia. What can be expected, however, and is supported by this study’s findings, is that the BFCN are altered in patients (along with other brain regions) and that they are associated with and even contribute to the patients’ attentional deficits.

We also tested whether the degree of smoothing might have an effect on the main results. Specifically, in the COBRE cohort, we smoothed the modulated images with a 4 mm instead of 8 mm FWHM and repeated the main analyses. We found that the level of smoothing did not affect the current results (see Supplementary Results and Fig. S3 for details). Furthermore, as ventricular enlargement is a common feature in patients with schizophrenia [95], we tested whether alterations to ventricular size drove the changes in BFCN. We found that, by controlling for ventricular size (which was increased in patients), the group difference in BFCN volumes was no longer significant (see Supplementary Results for details). However, changes in global GM were also no longer significant after controlling for ventricular size, consistent with the complex relationship between ventricular enlargement and GM volume loss in schizophrenia [96]. Although we cannot exclude that ventricular enlargement drives the group difference in BFCN volumes, we speculate that several factors might contribute to lower BFCN volumes, including increased ventricle size and decreased global GM volumes. Next, the sample sizes of the cohorts used in this study were relatively modest, therefore findings should be evaluated carefully as they may not be definitive. Finally, we did not find an effect of smoking on any of the main outcomes, but we cannot completely exclude an effect, as the groups were not properly matched in this regard.

## Conclusion

BFCN volumes are lower in schizophrenia, and this impairment links with patients’ attentional deficits. Data suggest that a dysregulated cholinergic system might contribute to cognitive difficulties in schizophrenia via impaired BFCN.

## Funding and disclosure

Acquisition and data analysis of the Munich cohort was supported by the European Union 7^th^ Framework Programme, TRIMAGE, a dedicated trimodality imaging tool for schizophrenia (Grant no. 602621). MJG is supported by the “Miguel Servet” program [CP19/00031] and a research grant [PI20/00613] of the Instituto de Salud Carlos III-Fondo Europeo de Desarrollo Regional (ISCIII-FEDER). The authors report no conflict of interest relating to this work. SL has received honoraria for consulting or lectures from LB Pharma, Lundbeck, Otsuka, TEVA, LTS Lohmann, Geodon Richter, Recordati, Boehringer Ingelheim, Sandoz, Janssen, Lilly, SanofiAventis, Servier, and Sunovion.

## Supplementary information

Supplemental Material
