# Supplementary material for: Lower cholinergic basal forebrain volumes link with cognitive difficulties in schizophrenia
Source: Neuropsychopharmacology. 2021 Jun 29;46(13):2320–9. doi: 10.1038/s41386-021-01070-x (PMC8580980; doi:10.1038/s41386-021-01070-x)
Supplement: Supplementary file 1 — Supplemental Material [file 41386_2021_1070_MOESM1_ESM.doc]

**Supplementary material for**

*Lower cholinergic basal forebrain volumes link with cognitive difficulties in schizophrenia*

By Avram et al.

Table of Contents

[**Supplementary Methods 2**](#__RefHeading___Toc73442047)

[Participants 2](#__RefHeading___Toc73442048)

[**The COBRE cohort** 2](#__RefHeading___Toc73442049)

[**MRI data acquisition in the COBRE cohort** 2](#__RefHeading___Toc73442050)

[**The Munich cohort** 2](#__RefHeading___Toc73442051)

[**MRI data acquisition in the Munich cohort** 3](#__RefHeading___Toc73442052)

[Mediation analysis 3](#__RefHeading___Toc73442053)

[Control and specificity analyses 4](#__RefHeading___Toc73442054)

[Control analysis on the group difference in BFCN volumes with a voxel-wise approach 7](#__RefHeading___Toc73442055)

[Control analyses for the specificity of BFCN sub-regions 7](#__RefHeading___Toc73442056)

[Control analyses for the effect of cognitive assessment 8](#__RefHeading___Toc73442057)

[Global GM influences on regional volume changes in patients 8](#__RefHeading___Toc73442058)

[Control for the effect of sex 10](#__RefHeading___Toc73442059)

[Control for the effect of age 10](#__RefHeading___Toc73442060)

[Control for the effect of smoking 11](#__RefHeading___Toc73442061)

[Control for the effect of ventricular enlargement 12](#__RefHeading___Toc73442062)

[Control analyses for the effect of positive symptoms on lower BFCN volumes 12](#__RefHeading___Toc73442063)

[Control for the level of smoothing 13](#__RefHeading___Toc73442064)

[Replication study in the Munich cohort 13](#__RefHeading___Toc73442065)

[Supplementary Discussion 17](#__RefHeading___Toc73442066)

[Confounding effects of global GM volume on BFCN findings 17](#__RefHeading___Toc73442067)

[Tables 20](#__RefHeading___Toc73442068)

[Table S1: Antipsychotic and adjunctive psychotropic medication in the COBRE cohort 20](#__RefHeading___Toc73442069)

[Table S2: Antipsychotic and adjunctive psychotropic medication in the Munich cohort 21](#__RefHeading___Toc73442070)

[Table S3: Percentiles of BFCN volumes and SCT scores in the COBRE cohort 22](#__RefHeading___Toc73442071)

[Figures 23](#__RefHeading___Toc73442072)

[Figure S1: Map of the cholinergic basal forebrain 23](#__RefHeading___Toc73442073)

[Figure S2: Voxel-wise control for BFCN reduction in patients in the COBRE cohort 24](#__RefHeading___Toc73442074)

[Figure S3: Voxel-wise control for the level of smoothing in the COBRE cohort 25](#__RefHeading___Toc73442075)

[Figure S4: Violin plots reflecting data distribution for BFCN volumes and SCT scores 26](#__RefHeading___Toc73442076)

[Figure S5: Voxel-wise relationship between whole-brain GM voxels and SCT scores 27](#__RefHeading___Toc73442077)

[References 28](#__RefHeading___Toc73442078)

# **Supplementary Methods**

## **Participants**

### **The COBRE cohort**

72 patients with schizophrenia meeting DSM-IV-TR criteria (age range: 18-65 years; mean: 38.16±13.89 years) and 73 healthy controls (age range: 18-65 years; mean: 35.60±11.50 years) from the COBRE dataset were included in this study. Schizophrenia was diagnosed according to DSM-IV–TR criteria on the basis of a structured clinical interview [1]. Patients’ medication was kept stable for at least 4 weeks prior to the study [2]. For antipsychotic and adjunctive psychotropic medication see Table S1. The healthy controls, matched for age and sex with the patient sample, had no history of DSM-IV Axis I disorders, or psychosis in any first-degree relatives. Substance abuse was eliminated via urine-screening. All subjects gave written informed consent before scanning sessions.

### **MRI data acquisition in the COBRE cohort**

MRI data acquisition was performed on a 3 T Siemens Trio MRI scanner with a 12-channel radio frequency coil. A high-resolution, anatomical T1-weighted MRI image was acquired with the following parameters: TR/TE/TI: 2530/(1.64, 3.5, 5.36, 7.22, 9.08)/900ms, flip angle: 7°, FoV: 256, matrix size: 256×256, voxel size: 1×1×1mm^3^. For more information see http://fcon_1000.projects.nitrc.org/indi/retro/cobre.html.

### **The Munich cohort**

We recruited 26 patients with schizophrenia meeting DSM-IV-TR criteria (age range: 23-65 years; mean: 42.84±11.38 years) from the Department of Psychiatry of Klinikum rechts der Isar, Munich. The patients recruited for the study had ‘established’ schizophrenia (i.e., at least two previous psychotic episodes) and were in symptomatic remission of psychotic symptoms at the time of the scan, in line with the criteria of Andreasen and colleagues [3]. Specifically, patients' scores on the Positive and Negative Syndrome Scale (PANSS) items ’delusions’ (P1), ‘conceptual disorganization’ (P2), 'hallucinatory behavior' (P3), 'mannerisms/posturing' (G5), and 'unusual though content' (G9) were ≤3. No other remission criteria had to be fulfilled. Antipsychotic medication was kept stable for minimum two weeks before the scan. See Table S2 for antipsychotic and adjunctive psychotropic medication. Twenty-four healthy controls (age range:25-62 years; mean: 38.54±11.63 years), comparable regarding age and sex with the patient sample, were also recruited from the same geographic area. The control subjects had no personal history of Axis I disorders, substance abuse, nor first-degree relatives with a history of psychosis. Substance abuse, in all participants, was eliminated via urine-screening or clinical interview. The study was approved by the Ethics Review Board of the Klinikum rechts der Isar of the Technical University Munich, and patients completed their written informed consent.

### **MRI data acquisition in the Munich cohort**

MRI data acquisition was performed with a hybrid whole-body mMR Biograph PET/MRI scanner (Siemens-Healthineers, Erlangen, Germany), with a vendor-supplied 12-channel phase-array coil. A high-resolution, anatomical T1-weighted MRI image was acquired with the following parameters: TR/TE/flip angle: 2300 ms/2.98 ms/9°; 160 slices (gap 0.5 mm) covering the whole brain; FoV: 256 mm; matrix size: 256×256; voxel size: 1×1×1mm^3^.

## **Mediation analysis**

To test whether the attentional deficits seen in patients were mediated by lower BFCN volumes, we performed mediation analysis with the PROCESS software package [4]. Typically, mediation analysis tests whether and how the effect of a causal variable (group) on an outcome variable (SCT) is affected by a mediator variable (BFCN volumes) [5]. In more detail, the relationship between such variables is described by three effects, displayed by a path diagram (Figure 2C): ‘total effect’ *c* – reflecting the ‘simple' association between causal variable and outcome variable; ‘direct effect’ *c'* – showing the effect of the causal variable on the outcome variable, when controlling for the mediator variable; and ‘indirect effect’ *ab* – showing the effect of the causal variable on the outcome variable via the mediator variable. Seven healthy controls were excluded from the model, as they had missing SCT scores. The statistical significance of the indirect effect, was tested with a nonparametric bootstrapping approach (5000 iterations) to obtain 95% confidence intervals [4]. To control for possible influences of global GM volumes and smoking on the relationship between BFCN volumes and group difference in SCT, we repeated the analysis and added global GM and smoking, respectively, as a covariates-of-no-interest in the models.

## **Control and specificity analyses**

To ensure that putative differences in BFCN volumes and their link to patients’ attentional deficits were not driven by some methodological artefact, and to better understand our results in terms of relative specificity, we performed several control and specificity analyses for the following factors: the effect of ROI-based versus voxel-based approaches, regional specificity for BFCN sub-regions, the influence of anticholinergic medication effects, the specificity of cognitive assessment, the specificity of global GM influence on regional volume changes, and the replicability of findings in other patient samples.

*(i) Voxel-wise approach on BFCN volume*.

Both to test for the potential effect of the ROI-based approach on group differences in BFCN volumes, and to locate peak differences in the BFCN, we performed a voxel-wise two-sample t-test restricted to the BFCN ROI with TIV as covariate-of-no-interest (p_FWE_=0.05) via SPM12 in the COBRE cohort.

*(ii)* *BFCN sub-regions*

Related to the previous point, we investigated the regional specificity of BFCN volume changes by investigating an alternative BFCN mask, generated based on a "consensus" of all available stereotactic BFCN maps to date [6-8], and consisting of an anterior and posterior cluster, based on functional parcellation [9], which we investigated separately due to their distinct projections and functional specializations [9, 10]. The anterior BFCN largely corresponds to the medial septal nucleus, diagonal band, and anterior-medial parts of the nucleus basalis of Meynert (NBM), whereas the posterior part covers the remaining neocortically-projecting part of the NBM. As for the composite BFCN, group differences in the anterior and posterior BFCN were evaluated with two-sample t-tests. Next, the association with SCT scores was evaluated for each cluster, separately, in patients. Finally, mediation analysis was used to evaluate whether anterior BFCN or posterior BFCN, respectively, mediate the group difference in SCT.

*(iii) Effect of anticholinergic burden of medication.*

Next, we investigated whether the association between BFCN volumes and SCT scores was influenced by the ACB of each medication quantified with the ACB scale [11, 12], using a non-parametric partial correlation approach (as ACB scores were not normally distributed, see below), with the ACB scores as covariates-of-no-interest. To calculate the ACB score for each participant, each drug was rated on a Likert scale ranging from 0-3, where 0 = no, 1 = mild, 2 = moderate, and 3 = severe anticholinergic activity. These scores were then summed for each patient into a final ACB score.

*(iv) Specificity of the link between BFCN volume changes and cognitive assessment*

(a) We investigated whether BFCN volumes were also related to SCT scores in healthy controls, akin to the evaluation in patients. (b) In addition, to evaluate whether BFCN volumes link specifically with attention or rather with the speed of processing, we also assessed the relationship between BFCN volumes and TMT-A scores in patients, using the same approach as for SCT.

*(v) Global GM influences on regional volume changes in patients*

To test for the relative specificity of lower BFCN volumes relative to global GM changes, we computed several control analyses, which included global GM as covariate-of-no-interest. To foreshadow results, global GM had an effect on all main analyses (i.e., BFCN volume difference, association between BFCN volumes and SCT, and mediation of BFCN of the group difference in SCT). To better understand this global GM influence, we tested whether global GM also influences volume changes of other brain regions in patients, and their relation with SCT. We focused on the anterior cingulate cortex (ACC) and insular cortices, regions that typically have lower volumes in schizophrenia and have been associated with attention and other cognitive processes [13-16], and the striatum and thalamus, as control regions. Specifically, we tested: (a) whether these ROIs had lower volumes in patients with schizophrenia compared to healthy controls, (b) in case of lower volumes, whether the group difference remained significant after controlling for global GM (i.e., volume specificity), (c) whether the ROIs correlated with SCT scores and mediated the group difference in SCT, and (d) in case of mediation, whether these analyses remained significant after controlling for global GM (i.e., functional specificity). We created the ROIs for the ACC and bilateral insula from the Harvard-Oxford cortical atlas, and for the striatum and thalamus from the Harvard-Oxford subcortical atlas in FSLeyes. Volumes were computed as for BFCN and normalized for TIV.

*(vi) Replicability of findings and control for the effect of psychosis*

Structural MRI data were analyzed with VBM analogously to the COBRE cohort. Specifically, we investigated group differences in BFCN volumes, and subsequently, studied the link between BFCN volumes and SCT performance with correlation and mediation analyses.

**Supplementary Results**

## **Control analysis on the group difference in BFCN volumes with a voxel-wise approach**

To control for the regional, ROI-based approach, and to locate peak differences between patients and healthy controls in BFCN in the COBRE cohort, we employed a two-sample voxel-wise t-test restricted to the BFCN ROI in SPM12 (cluster-level p_FWE_=0.05), using TIV as covariate-of-no-interest. Gray matter volumes of the BFCN were reduced in patients (t(142)=3.53, p_FWE_<0.05), and peaked in the medial septum (x=0, y=0, z=-6) (Figure S2).

## **Control analyses for the specificity of BFCN sub-regions**

In order to test for specificity of BFCN sub-regions, we investigated another mask of the BFCN, consisting of an anterior and posterior cluster, based on functional parcellation [9]. Regarding the anterior BFCN, we found lower volumes in patients compared to healthy controls, as shown by a two-sample t-test (t(139)=2.71, p=0.007). In patients, lower anterior BFCN volumes were positively associated with SCT scores, as demonstrated by Pearson correlation analysis (r=0.31, p=0.01). Finally, mediation analysis demonstrated that anterior BFCN volumes mediated the group difference in SCT scores (ab=-1.10±0.62; the bootstrapped 95% confidence interval CI[-2.51 -0.10]). Correspondingly, concerning the posterior BFCN, we found lower volumes in patients compared to healthy controls, as shown by a two-sample t-test (t(139)=2.01, p=0.04). In patients, lower posterior BFCN volumes were positively associated with SCT scores, as demonstrated by Pearson correlation analysis (r=0.38, p=0.002). Finally, mediation analysis demonstrated that posterior BFCN volumes mediated the group difference in SCT scores (ab=-1.39±0.70; the bootstrapped 95% confidence interval CI[-2.94 -0.19]). We conclude that although the group difference in BFCN volumes appeared somewhat more accentuated in the anterior BFCN (in line with the voxel-based analysis), and the association with attentional scores more prominent for the posterior BFCN, the findings were not specific to either cluster.

## **Control analyses for the effect of cognitive assessment**

To test whether lower BFCN volumes linked specifically to speed of processing information, or rather attentional processing, we also investigated the association with an additional cognitive test, namely the Trail Making Test Part A (TMT-A), which mainly measures speed of processing [17]. We first investigated whether TMT-A scores were normally distributed in the patient group of the COBRE cohort. A Shapiro-Wilk test demonstrated that the data were not normally distributed (p<0.001). We used Spearman correlation analysis to test for the association and found that the variables were not significantly correlated (rho=-0.16, p=0.20). We then repeated the approach for the Munich cohort. TMT-A scores were not normally distributed in the patient group of the Munich cohort (p=0.001). Therefore, Spearman correlation was used to assess the association between BFCN volumes and TMT-A scores. The relationship was not significant (rho=-0.31, p=0.11). As the correlation was not significant neither for the patients in the COBRE nor for the ones in the Munich cohort, we interpreted this finding, together with the association between lower BFCN volumes and SCT scores, as a specific link between lower BFCN volumes and diminished attentional capacity in patients, considering that the SCT assesses both attention and speed processing.

## **Global GM influences on regional volume changes in patients**

We found that global GM changes affected all main results regarding BFCN volumes, suggesting that global GM changes influence the effects seen for BFCN. We tested this hypothesis by analyzing the relative specificity relative to global GM of additional ROIs, namely the ACC, insula, thalamus, and striatum.

Regarding differences in ROI volumes, two-sample t-tests demonstrated that ACC volumes were lower in patients compared to healthy controls (t(139)=3.13, p=0.002), as were insular (t(139)=3.20, p=0.002), and thalamic volumes (t(139)=3.46, p=0.001), but not striatal ones (t(139)=-0.11, p=0.91). These findings are in line with previous reports of lower cortical and subcortical GM volumes in schizophrenia. Next, we checked for the relative specificity of volume group differences by conducting ANCOVAs for these ROIs with global GM as covariate-of-no-interest. ANCOVA demonstrated that when controlling for global GM the group differences were no longer significant neither for ACC (F_1,138_=0.80, p=0.37), nor for the insular volumes (F_1,138_=1.08, p=0.30), demonstrating that even very robust findings such as lower ACC and insular volumes in schizophrenia are affected by global GM changes. In contrast, we found a trend for significance for thalamic volumes (F_1,138_=3.59, p=0.06), indicating a somewhat weaker effect of global GM on the thalamus.

Next, we tested whether the ROIs correlated with SCT scores and whether they mediated the group difference in SCT. Pearson correlation analysis demonstrated a significant correlation between ACC volumes and SCT scores (r=0.40, p=0.001), and for insular volumes and SCT scores (r=0.30, p=0.01), but not for thalamic volumes and SCT scores (r=0.16, p=0.19), and striatal volumes and SCT scores (r=0.19, p=0.11) in patients. Remarkably, using the same partial correlation approach as for the BFCN, correlations between ACC/ insular volumes and SCT scores were no longer significant in patients (r=0.07, p=0.58 and r=-0.12, p=0.31, respectively), when controlling for global GM, indicating that lower global GM volumes influence the association between SCT scores and lower BFCN, ACC, and insular volumes, respectively. Furthermore, mediation analysis demonstrated that the indirect effect of ACC volumes on the group difference in SCT was significant (ab=-1.45±0.72; the bootstrapped 95% confidence interval CI[-3.10 -0.27]), as was the indirect effect of insular volumes on the group difference in SCT (ab=-1.71±0.74; the bootstrapped 95% confidence interval CI[-3.40 -0.47]). These results are in line with previous findings linking the ACC and insular cortices to cognitive process such as attention in schizophrenia [13, 15, 16].

Finally, we tested whether global GM had on effect on the ACC and insular volumes’ mediation of the group differences in SCT scores, respectively. We found the mediation of ACC volumes on the group difference in SCT no longer significant after controlling for global GM (ab=-0.03±0.17; the bootstrapped 95% confidence interval CI[-0.33 0.45]). Correspondingly, we also found the mediation of insular volumes on the group difference in SCT no longer significant after controlling for global GM (ab=-0.09±0.28; the bootstrapped 95% confidence interval CI[-0.41 0.77]). These results suggest that while both ACC and insular volumes are related to attentional processes, global GM modulates these relationships. Indeed, we found that global GM also mediates the group difference in SCT scores (ab=-2.08±0.89; the bootstrapped 95% confidence interval CI[-4.07 -0.54]). We therefore tested for global GM’s relative specificity in this mediation with regard to the ROIs that also mediated the group difference in SCT (i.e., BFCN, ACC, and insular volumes). The indirect effect of global GM was no longer significant when controlling for BFCN volumes (ab=-1.04±0.74; the bootstrapped 95% confidence interval CI[-2.81 0.12]), or for ACC volumes (ab=-0.03±0.17; the bootstrapped 95% confidence interval CI[-0.33 0.45]), or insular volumes (ab=-0.09±0.28; the bootstrapped 95% confidence interval CI[-0.41 0.77]). These findings indicate a complex relationship between global GM and ROIs relevant for attentional deficits, suggesting that there is not one region that drives the association with attention, but rather that several brain regions might contribute to such an effect.

For replication purposes, we also tested for group differences in the amygdala and hippocampus, since these regions are also typically reported to have lower volumes in schizophrenia [13, 14]. Patients with schizophrenia had significantly lower volumes in both amygdala (t(139)=4.06, p<0.001) and hippocampus (t(139)=4.10, p<0.001).

## **Control for the effect of sex**

To test whether the marginal difference found in sex influences the group difference in BFCN volumes, we repeated the ANCOVA but used sex instead of global GM/WM as covariate-of-no-interest. The group difference remained significant (F_1,138_=4.92, p=0.02), indicating that sex does not influence the group difference in BFCN volumes.

## **Control for the effect of age**

Since age has an effect on GM volume, we tested whether it affects the group difference in BFCN volumes. We investigated this putative effect with ANCOVA, which included age as a covariate-of-no-interest. The group difference remained significant (F_1,138_=5.30, p=0.02). This finding indicates that the lower BFCN volumes seen in patients are not confounded by age.

## **Control for the effect of smoking**

Considering the high comorbidity between schizophrenia and smoking, the effects of smoking on ACh transmission, on GM, and on cognition, we tested whether smoking affected the main results of our study. We included smoking as a dummy variable (0 for non-smoker and 1 for smoker), and tested for effects in the COBRE cohort. Data was missing for one healthy control. Specifically, we included smoking as a covariate in the analysis concerning (i) group differences in BFCN volumes, (ii) associations between BFCN volumes and SCT scores, and (iii) mediation analysis investigating whether the group difference in SCT scores is mediated by lower BFCN volumes. Regarding (i), we investigated whether smoking affects the group difference in BFCN volumes via ANCOVA, which included smoking as a covariate-of-no-interest. The group difference remained significant (F_1,137_=4.38, p=0.03). This finding indicates that the lower BFCN volumes seen in patients are not explained by smoking status. Regarding (ii), we then tested whether smoking might have an effect on the relationship between lower BFCN volumes and lower SCT scores in patients. A Shapiro-Wilk test demonstrated that smoking was not normally distributed (p=0.001). We therefore computed a Spearman partial correlation which demonstrated that the association between BFCN volumes and SCT scores remained significant (rho=0.33, p=0.007) in patients. Regarding (iii), we tested whether BFCN volume mediation of the group difference in SCT scores was affected by smoking by including smoking as a covariate-of-no-interest in the model. The indirect effect remained significant (ab=-0.78±0.50; the bootstrapped 95% confidence interval CI[-0.94 -0.02]), indicating that the mediation is independent of smoking status.

## **Control for the effect of ventricular enlargement**

As ventricular enlargement is one of the most robust findings in schizophrenia [18], and could affect BFCN volumes, we tested whether putative ventricular enlargement could affect our main result in the COBRE cohort. We computed the volumes of the lateral ventricles in the same manner as for the other ROIs examined in this paper. Briefly, we used a (bilateral) lateral ventricle mask, created in FSLeyes from the Harvard-Oxford subcortical atlas, to extract values from the modulated and warped CSF maps. The ventricular volumes were then normalized to TIV. A two-sample t-test demonstrated that ventricles were enlarged in the patients with schizophrenia of the COBRE cohort compared to the healthy controls (t(139)=-3.38, p=0.001). Furthermore, there was a significant negative correlation between ventricular volumes and BFCN volumes in patients (r=-0.34, p=0.004). The group difference in BFCN volumes was no longer significant after controlling for ventricular size, as shown by ANCOVA (F_1,138_=2.16, p=0.14). This finding indicates that ventricular enlargement might drive the group difference seen in BFCN volumes in schizophrenia. However, in light of the fact that evidence suggests a complex relationship between ventricular enlargement and GM volume loss in schizophrenia [19], we tested whether the group difference in global GM would remain significant after controlling for ventricular size, as global GM and ventricular volumes were also significantly correlated in patients (r=-0.544, p<0.001). ANCOVA demonstrated that groups did not differ in global GM after controlling for ventricular volumes (F_1,138_=2.58, p=0.11). Taken together, it is possible that ventricular enlargement might drive the group difference seen in BFCN volumes, however, this might be an unspecific effect, related rather to the relationship between global GM volumes and ventricular size.

## **Control analyses for the effect of positive symptoms on lower BFCN volumes**

No associations were found between BFCN volumes and psychotic symptoms (as measured by PANSS positive scale) with Pearson correlation analyses in neither COBRE (r=-0.11, p=0.36) nor Munich cohort (r=0.11, p=0.59).

## **Control for the level of smoothing**

Considering the small size of the BFCN region we tested whether the level of smoothing could affect the results. We performed a control analysis in the COBRE cohort, smoothed the modulated images with a 4 mm FWHM and repeated the main analyses, namely the ROI-based analysis regarding BFCN volume differences, the association between BFCN volumes and SCT scores, and the mediation of the group differences in SCT via BFCN volumes. In addition, we also computed a voxel-wise analysis on the 4mm smoothed data, restricted to the BFCN region. First, regrading volume differences, we found the BFCN volumes to be lower in patients, via a two-sample t-test (t(139)=2.09, p=0.03). Second, Pearson correlation analysis showed that BFCN volumes were significantly associated with SCT scores in patients (r=0.36, p=0.003). Third, mediation analysis demonstrated that BFCN volumes significantly mediated the group difference in SCT scores (ab=-1.16±0.66; the bootstrapped 95% confidence interval CI[-2.70 -0.14]). Finally, voxel-wise volume analysis, restricted to the BFCN region, demonstrated lower BFCN volumes in patients, peaking in the medial septum (x=0, y=0, z=-6) (Figure S3). In light of these findings, we conclude that the level of smoothing did not affect the current results.

## **Replication study in the Munich cohort**

To assess the replicability of our results in the COBRE cohort, we used the identical approach in the independent Munich cohort. Briefly, we investigated whether (i) BFCN volumes were also lower in patients, (ii) BFCN volume alterations are associated with patients’ performance on SCT, and finally (iii) lower BFCN volumes mediate the group difference in SCT.

First, we tested for group differences in BFCN volumes. In more detail, a two-sample test on BFCN volumes demonstrated a significant reduction in patients (t(48)=2.05, p=0.04; Figure 3A). Next, we tested whether the lower BFCN volumes observed in patients were potentially influenced by other variables. We started by investigating differences in global GM and WM. Two-sample t-tests demonstrated that patients differed from healthy controls in global GM (t(48)=2.90, p=0.006) – i.e., had lower volumes, but not in global WM (t(48)=-0.46, p=0.64). To test whether global WM or GM volume might influence the group difference in BFCN volumes, we computed two ANCOVAs with global WM and GM volume as covariate-of-no-interest, respectively. The group difference in BFCN volumes remained significant when controlling for global WM volume (F_1,47_=4.08, p=0.04), indicating that the group difference was not influenced by global WM volume. However, the group difference in BFCN volumes was no longer significant after controlling for global GM volume (F_1,47_=0.01, p=0.9), indicating that lower global GM volumes influence the group difference in BFCN volumes. This result is in line with the findings in the COBRE cohort.

Next, we found that current medication (CPZ) was not associated with lower BFCN volumes, as demonstrated by Spearman’s correlation analysis (rho=-0.04, p=0.82).

Second, we investigated the associations between lower BFCN volumes and patients’ performance on SCT. A Shapiro-Wilk test demonstrated that the SCT scores were normally distributed in the patient group (p=0.5). Pearson’s correlation analysis demonstrated a marginally significant association between lower BFCN volumes and SCT scores in patients (r=0.36, p=0.07; Figure 3B). As groups differed in global GM volume, we tested whether global GM volumes influenced the relation between lower BFCN and attentional deficits in patients. Initially, we investigated whether global GM volumes were associated with SCT scores in patients, using Pearson correlation analysis. We found that SCT scores correlated with global GM (r=0.62, p=0.001). To test whether the association between BFCN volumes and SCT scores was influenced by global GM volume, we computed a partial correlation analysis between BFCN volumes and SCT scores in patients, controlling for global GM volume. The relationship between BFCN volumes and SCT scores was no longer significant in patients after controlling for global GM volume (r=-0.11, p=0.57), indicating that the correlation between BFCN volumes and SCT scores in patients was influenced by global GM volume. This result is also in line with the findings reported for the COBRE cohort.

To control for the putative effect of ACB of medication on this association, we computed a partial correlation with ACB scores as covariate-of-no-interest (see Table S2 for ACB scores). A Shapiro-Wilk test demonstrated that ACB scores were not normally distributed (p<0.001). We therefore computed a Spearman partial correlation, to investigate the effect of ACB on the relationship between BFCN volumes and SCT scores. The association between BFCN volumes and SCT scores remained borderline significant (rho=0.27, p=0.05), indicating a minor effect of ACB on this association.

Third, we further analyzed the link between BFCN volume alterations and attentional performance, by investigating whether lower BFCN volumes mediate the group effect on SCT via mediation analysis. We first investigated the link between BFCN volumes and SCT scores in healthy controls. A Shapiro-Wilk test demonstrated that SCT scores were normally distributed in the healthy control group (p=0.34). No significant correlation was found between BFCN volumes and SCT scores (r=0.30, p=0.14), as shown by Pearson correlation analysis. Mediation analysis, revealed a significant indirect effect of group on SCT via lower BFCN volumes (ab=-2.35±1.50; the bootstrapped 95% confidence interval CI[-5.84 -0.04]; Figure 3C), indicating a significant mediation of the group difference in SCT (see Table 1) via lower BFCN volumes. We conclude that lower BFCN volumes likely contribute to attentional deficits in patients with established schizophrenia. To control for the putative effect of lower global GM volumes on the mediation of the group difference in SCT via lower BFCN volumes, we repeated the mediation analysis but included global GM volume as a covariate-of-no-interest in the model. The indirect effect was no longer significant (ab=0.03±0.45; the bootstrapped 95% confidence interval CI[-0.78 1.14]), indicating that lower global GM volume influences the mediation of lower BFCN volumes on the group difference in SCT performance. Akin to the COBRE cohort, we found that global GM volume also mediated the group difference in SCT scores (ab=-5.69±2.61; the bootstrapped 95% confidence interval CI[-11.81 -1.61]), but that this mediation was no longer significant after controlling for BFCN (ab=0.03±0.45; the bootstrapped 95% confidence interval CI[-0.78 1.14]). These results indicate that while lower global GM volumes are related to attentional deficits in schizophrenia, this effect is not independent of BFCN volumes. These results replicate the findings reported for the COBRE cohort.

# **Supplementary Discussion**

## **Confounding effects of global GM volume on BFCN findings**

Control analyses demonstrated a clear and consistent influence of global GM volume, on all main outcomes of the study in both cohorts: (i) the group difference in BFCN volumes, (ii) the associations between BFCN and SCT, and (iii) the mediation of the group difference in SCT via BFCN volumes. This influence of global GM volume on the main results was also found in the validation sample. These results indicate a twofold influence of global GM volume: (a) lower global GM influences – or maybe more precise – co-varies with the regional differences found for the “attentional ROIs” and (b) lower global GM co-varies with the group difference in attentional performance.

(a) Volume differences. As controlling for global GM volume rendered the group difference in BFCN volumes no longer significant, it appears that schizophrenia does not affect BFCN volumes more than the volumes of other regions, rather we are presented with a non-specific, distributed effect of lower overall GM. This is an important finding, and it is interesting that it extends to other areas typically found to be affected in schizophrenia (e.g., ACC, insula). While it is possible that all investigated regional differences were driven by lower global GM volumes, we consider it somewhat unlikely that the investigated ROIs themselves were unaffected. Lower volumes in the ACC and insula are one of the most consistent volumetric findings in schizophrenia [13, 14], and there is evidence that these regions have lower volumes in schizophrenia even when patients do not differ from healthy controls in global GM, and global GM is controlled for [20]. However, we cannot completely exclude the possibility that the group difference found for BFCN was influenced by the patients’ lower global GM volumes. Future studies might clarify this issue by investigating BFCN volumes in patients with schizophrenia whose global GM volumes do not differ from those of healthy controls. As GM changes appear to be more pronounced in chronic patients, perhaps patients with first-episode schizophrenia would make a good target group [20, 21].

(b) Relevance for attentional performance. The finding that controlling for global GM volume renders the mediation of the group difference in attentional performance via lower BFCN volumes no longer significant, suggests that not the BFCN in particular, but rather smaller global GM volumes are related to poor attentional performance. Although a limitation, this finding begs the question why global GM would have such an effect and whether we can disentangle it from that of BFCN? Disentangling the effect of global GM volume from that of “attentional ROIs” on attentional task performance in general is a complex issue, as brain regions that typically present with lower volumes in chronic schizophrenia overlap with regions implicated in attentional processes (e.g., ACC, insula, frontal cortices etc.) [13-16]. Furthermore, it makes sense conceptually that complex cognitive processes such as attention are not specifically related to a single region but rather to several regions, which are functionally linked/ organized into networks (e.g., the salience network) [15, 16]. Indeed, there is evidence that BFCN or at least subdivisions of the BFCN are functionally coupled (i.e., via intrinsic functional connectivity) with some brain networks involved in attentional processes (i.e., salience network) [22]. As there are several attention-relevant networks (i.e., so-called salience, frontoparietal, dorsal attentional network) that cover extensive areas of global GM, it is perhaps not surprising that global GM volume is associated with attention and explains more variance than a singular region. Unfortunately, the analyses used in this study are not well suited to investigate such a distributed effect. However, we tried to tackle this issue with an additional control analysis. Specifically, we computed a whole-brain analysis, in which multiple regression was used to investigate the relationship between each gray matter voxel and SCT performance in the patients of the COBRE cohort. To identify the effects of global GM, we ran the analysis twice, one controlling only for TIV, and once controlling only for global GM. The first analysis revealed very similar results with the ROI-based analyses, with clusters of voxels in the BFCN, ACC, and insula correlating with SCT scores in patients (Figure S5). However, also in line with the ROI analyses, these effects disappeared when controlling for global GM instead of TIV.

A possible solution to disentangle the effect of global GM from that BFCN might be to employ multivariate approaches, in which the participants’ gray matter is parcellated into several regions and the relationship between these volumes and one or more attentional scores is investigated simultaneously, for instance via multivariate pattern analysis [23]. Future studies might also be inclined to investigate BFCN alterations and their relevance for attention in schizophrenia via resting-state functional connectivity, as functional connectivity is relatively independent of gray matter.

# **Tables**

**Table S1:** Antipsychotic and adjunctive psychotropic medication in the COBRE cohort

| **Antipsychotic medication** | **ACB score** | **Number of patients (N = 72)** |
| --- | --- | --- |
| Aripiprazole | 1 | 11 |
| Clozapine | 3 | 11 |
| Fluphenazine | 0 | 1 |
| Haloperidol | 1 | 7 |
| Olanzapine | 3 | 11 |
| Perphenazine | 1 | 1 |
| Quetiapine | 3 | 12 |
| Risperidone | 1 | 26 |
| Thiothixene | 0 | 1 |
| Ziprasidone | 1 | 7 |
| **Adjunctive psychotropic medication** |  | |
| Ativan | 0 | 8 |
| Benztropine | 3 | 6 |
| Bupropion | 1 | 3 |
| Carbamazepine | 2 | 1 |
| Citalopram | 1 | 3 |
| Clonazepam | 0 | 3 |
| Diazepam | 1 | 1 |
| Divalproex | 1 | 2 |
| Escitalopram | 1 | 1 |
| Fluoxetine | 0 | 3 |
| Fluvoxamine | 1 | 1 |
| Mirtazapine | 0 | 1 |
| Propranolol | 1 | 6 |
| Sertraline | 0 | 8 |
| Trazodone | 1 | 4 |
| Venlafaxine | 1 | 1 |
| Zaleplon | 0 | 1 |
| Zolpidem | 1 | 2 |

Current antipsychotic and adjunctive psychotropic medication for patients with schizophrenia. ACB – anticholinergic burden of medication.

**Table S2:** Antipsychotic and adjunctive psychotropic medication in the Munich cohort

| **Antipsychotic medication** | **ACB score** | **Number of patients (N = 26)** |
| --- | --- | --- |
| Amisulpride | 1 | 3 |
| Aripiprazole | 1 | 7 |
| Clozapine | 3 | 3 |
| Flupentixol | 1 | 3 |
| Haloperidol | 1 | 1 |
| Olanzapine | 3 | 9 |
| Paliperidone | 1 | 2 |
| Perazine | 1 | 1 |
| Perphenazine | 1 | 1 |
| Pipamperone | 1 | 1 |
| Quetiapine | 3 | 4 |
| Risperidone | 1 | 3 |
| **Adjunctive psychotropic medication** |  | |
| Biperiden | 3 | 3 |
| Bupropion | 1 | 1 |
| Citalopram | 1 | 3 |
| Duloxetine | 1 | 1 |
| Escitalopram | 1 | 1 |
| Methylphenidate | 1 | 1 |
| Mirtazapine | 0 | 1 |
| Lithium | 1 | 2 |
| Opipramol | 2 | 1 |
| Sertraline | 0 | 3 |
| Trimipramine | 3 | 1 |
| Valrpoate | 1 | 1 |
| Venlafaxine | 1 | 2 |
| Zolpidem | 1 | 1 |

Current antipsychotic and adjunctive psychotropic medication for patients with schizophrenia. ACB – anticholinergic burden of medication.

## **Table S3:** Percentiles of BFCN volumes and SCT scores in the COBRE cohort

|  | | **Percentiles** | | |
| --- | --- | --- | --- | --- |
|  | | **25%** | **50%** | **75%** |
| **BFCN**  **mm^3^** | HC | 281 | 296 | 315 |
|  | SCZ | 267 | 289 | 309 |
| **SCT**  **a.u.** | HC | 55 | 62 | 69 |
|  | SCZ | 39 | 44 | 53 |

Abbreviations: SCZ – patients with schizophrenia, HC – healthy controls, SCT – symbol coding task, BFCN – intracranial-normalized basal forebrain cholinergic nuclei.

**Figures**

## **Figure S1:** Map of the cholinergic basal forebrain


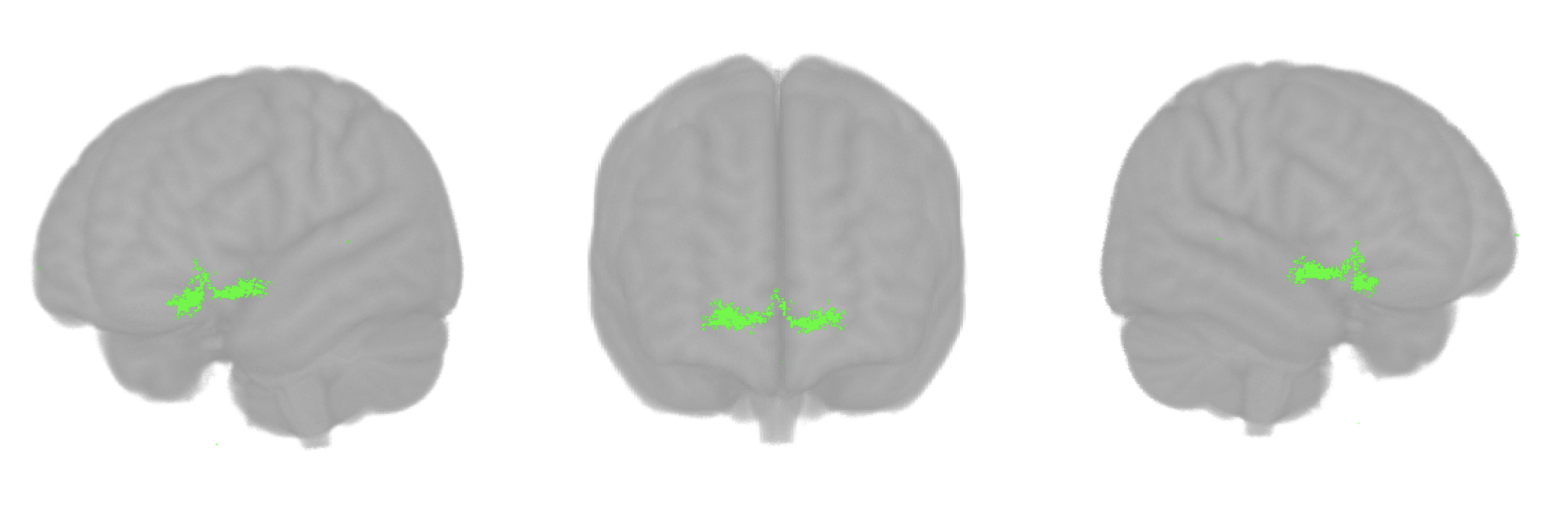


The region of interest depicts the cholinergic basal forebrain nuclei, based on a cytoarchitectonic map, overlaid on transparent renderings of the human brain in Montreal Neurological Institute space. The BFCN mask is based on combined histology and post-mortem MRI [7], containing several cholinergic subdivisions within the basal forebrain, including the medial septal nucleus, diagonal band of Broca, nucleus subputaminalis, the basal magnocellular complex, and nucleus basalis of Meynert [8, 24].

## **Figure S2:** Voxel-wise control for BFCN reduction in patients in the COBRE cohort


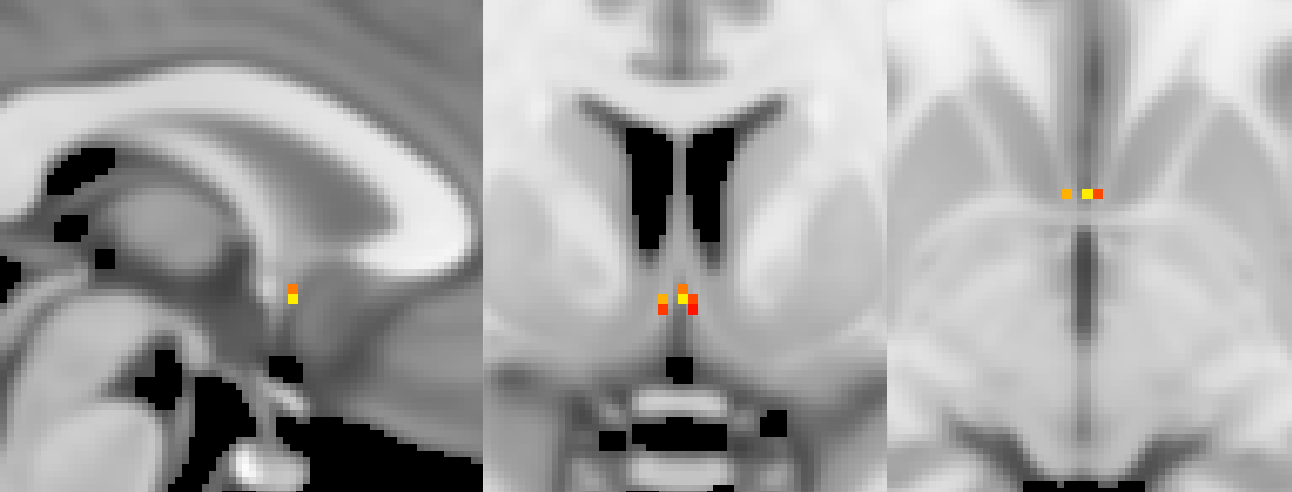


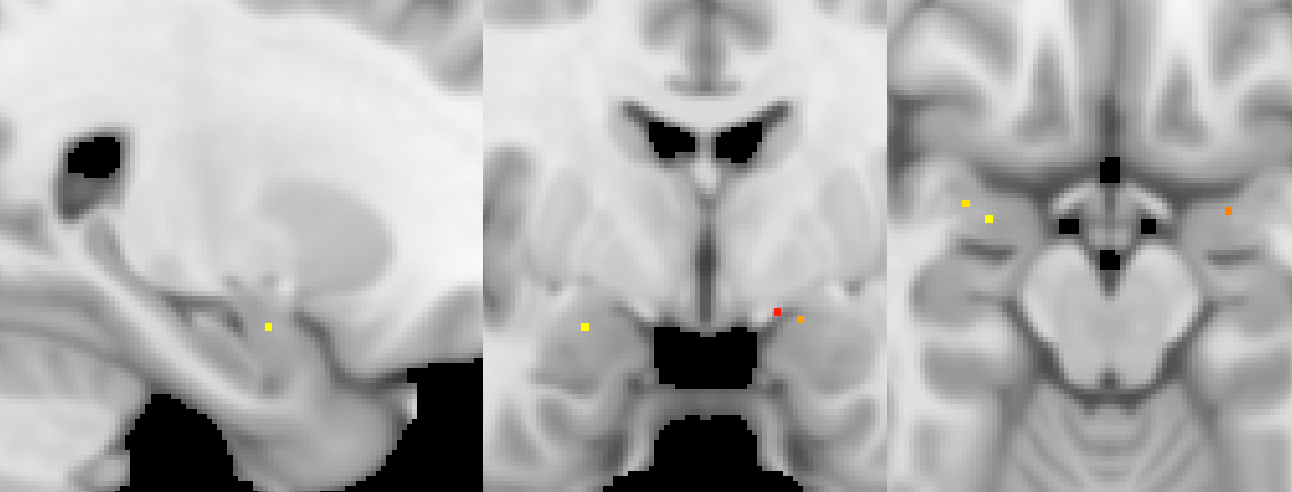


*Top*: the peak of the group difference in BFCN GM volumes is depicted. BFCN GM volumes were reduced in patients (t(115)=3.53, p_FWE_<0.05), and peaked in the medial septal nucleus (x=0, y=6, z=-6). *Bottom*: widespread volume reduction across the BFCN ROI is depicted. Results are based on a two-sample voxel-wise t-test restricted to the BFCN ROI, computed in SPM12 (voxel-wise p_FWE_=0.05), using TIV as covariate-of-no-interest.

## **Figure S3:** Voxel-wise control for the level of smoothing in the COBRE cohort

*
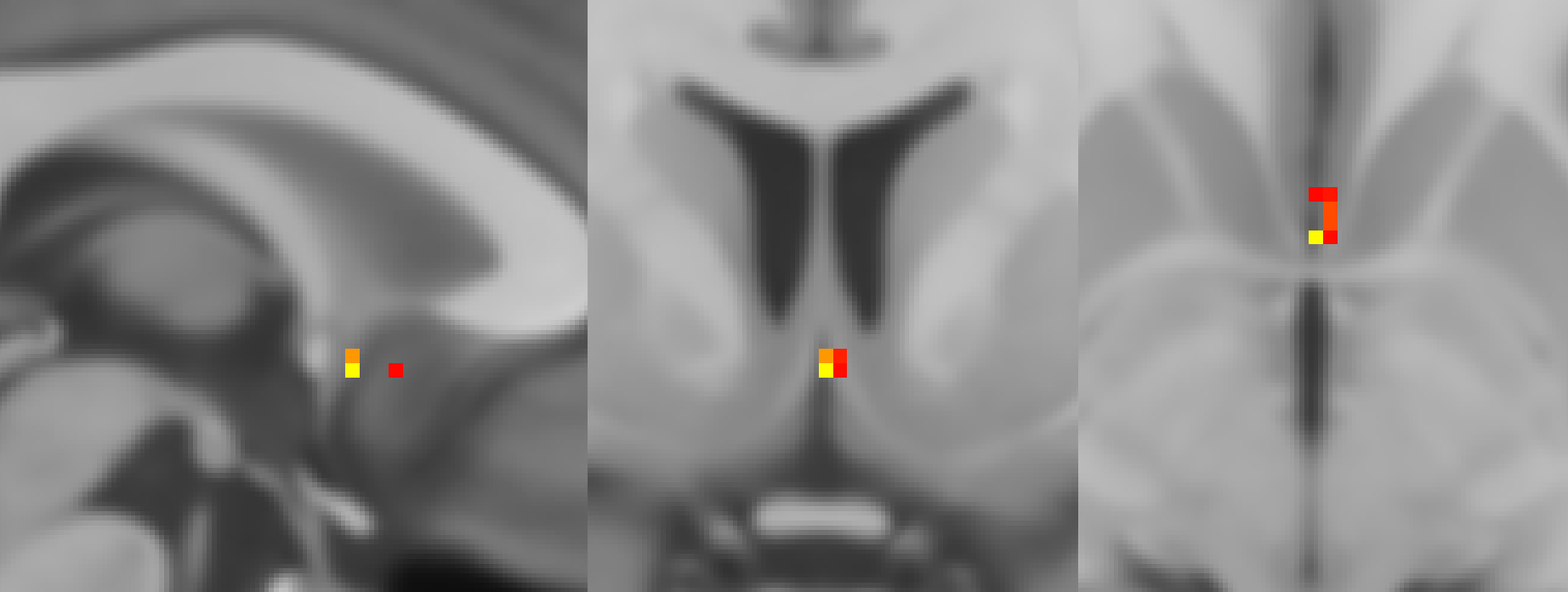
*

The peak of the group difference in BFCN GM volumes is depicted. BFCN GM volumes were reduced in patients (t(115)=3.53, p<0.001), and peaked in the medial septal nucleus (x=0, y=6, z=-6). Results are based on a two-sample voxel-wise t-test restricted to the BFCN ROI, computed in SPM12 (cluster-wise p_FWE_=0.05), using TIV as covariate-of-no-interest.

## **Figure S4:** Violin plots reflecting data distribution for BFCN volumes and SCT scores

*
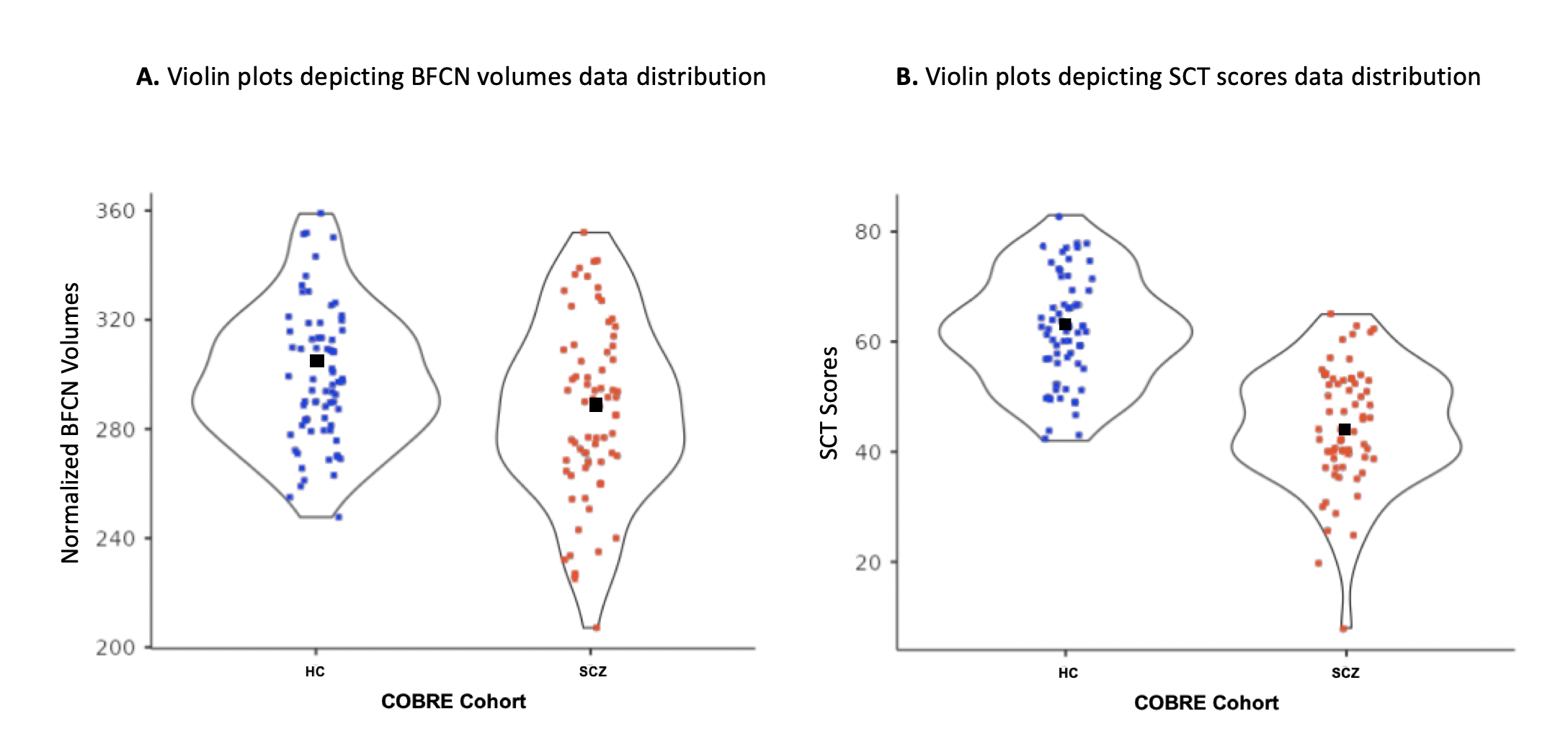
*

Abbreviations: SCZ – patients with schizophrenia, HC – healthy controls, SCT – symbol coding task, BFCN – intracranial-normalized basal forebrain cholinergic nuclei. Black squares depict the mean.

## **Figure S5:** Voxel-wise relationship between whole-brain GM voxels and SCT scores

*
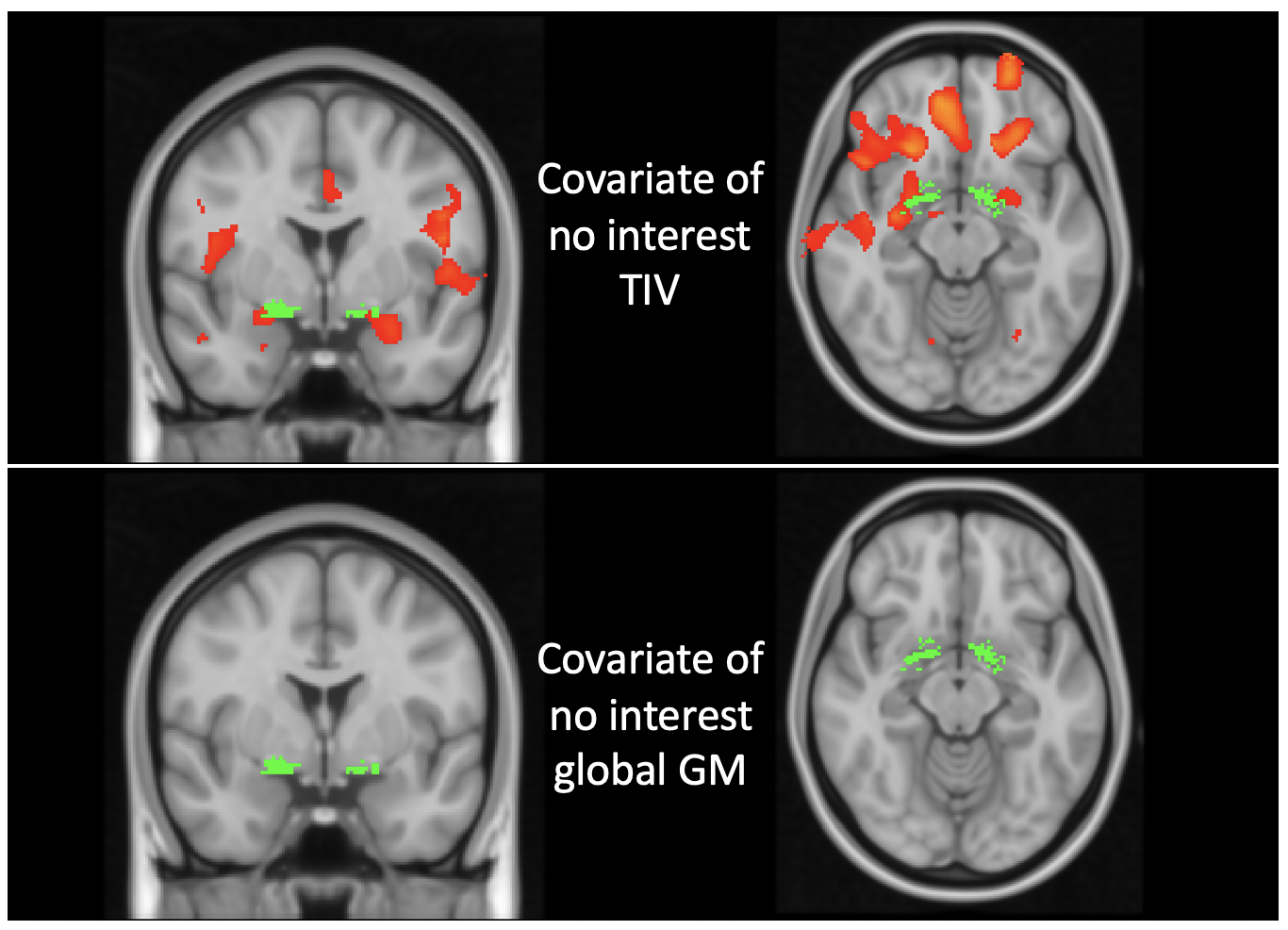
*

Multiple regression analyses investigating the relationship between SCT scores and all gray matter voxels in the patient group of the COBRE cohort. The BFCN mask (green) is overlaid for visualization purposes. *Top*: results are shown for a model, in which TIV is included as covariate-of-no-interest. *Bottom*: results are shown for an alternative model, in which global GM is included as covariate-of-no-interest. Significant clusters (orange-yellow) depict positive correlations between gray matter voxels and SCT scores. The analysis was computed in SPM12 (p<0.001, cluster level corrected p_FWE_=0.05).

# **References**

1. First MB, Spitzer RL, Gibbon M, Williams JBW. Structured Clinical Interview for DSM-IV-TR Axis I Disorders, Research Version, Patient Edition (SCID-I/P). New York: Biometrics Research, New York State Psychiatric Institute; 2002.

2. Calhoun VD, Sui J, Kiehl K, Turner J, Allen E, Pearlson G. Exploring the psychosis functional connectome: aberrant intrinsic networks in schizophrenia and bipolar disorder. Front Psychiatry. 2011;2:75. doi:10.3389/fpsyt.2011.00075

3. Andreasen NC, Carpenter WT, Jr., Kane JM, Lasser RA, Marder SR, Weinberger DR. Remission in schizophrenia: proposed criteria and rationale for consensus. Am J Psychiatry. 2005;162(3):441-9. doi:10.1176/appi.ajp.162.3.441

4. Hayes AF. Introduction to mediation, moderation, and conditional process analysis : a regression-based approach. New York: The Guilford Press; 2013.

5. Hayes AF, Rockwood NJ. Regression-based statistical mediation and moderation analysis in clinical research: Observations, recommendations, and implementation. Behav Res Ther. 2017;98:39-57.

6. Teipel SJ, Flatz WH, Heinsen H, Bokde AL, Schoenberg SO, Stockel S, et al. Measurement of basal forebrain atrophy in Alzheimer's disease using MRI. Brain. 2005;128(Pt 11):2626-44. doi:10.1093/brain/awh589

7. Zaborszky L, Hoemke L, Mohlberg H, Schleicher A, Amunts K, Zilles K. Stereotaxic probabilistic maps of the magnocellular cell groups in human basal forebrain. Neuroimage. 2008;42(3):1127-41. doi:10.1016/j.neuroimage.2008.05.055

8. Kilimann I, Grothe M, Heinsen H, Alho EJ, Grinberg L, Amaro E, Jr., et al. Subregional basal forebrain atrophy in Alzheimer's disease: a multicenter study. J Alzheimers Dis. 2014;40(3):687-700. doi:10.3233/jad-132345

9. Fritz HJ, Ray N, Dyrba M, Sorg C, Teipel S, Grothe MJ. The corticotopic organization of the human basal forebrain as revealed by regionally selective functional connectivity profiles. Hum Brain Mapp. 2019;40(3):868-78. doi:10.1002/hbm.24417

10. Ballinger EC, Ananth M, Talmage DA, Role LW. Basal Forebrain Cholinergic Circuits and Signaling in Cognition and Cognitive Decline. Neuron. 2016;91(6):1199-218. doi:10.1016/j.neuron.2016.09.006

11. Boustani M, Campbell N, Munger S, Maidment I, Fox C. Impact of anticholinergics on the aging brain: a review and practical application. Aging Health. 2008;4:311-20.

12. Salahudeen MS, Duffull SB, Nishtala PS. Anticholinergic burden quantified by anticholinergic risk scales and adverse outcomes in older people: a systematic review. BMC Geriatrics. 2015;15(1):31. doi:10.1186/s12877-015-0029-9

13. Goodkind M, Eickhoff SB, Oathes DJ, Jiang Y, Chang A, Jones-Hagata LB, et al. Identification of a common neurobiological substrate for mental illness. JAMA Psychiatry. 2015;72(4):305-15. doi:10.1001/jamapsychiatry.2014.2206

14. Brandl F, Avram M, Weise B, Shang J, Simoes B, Bertram T, et al. Specific Substantial Dysconnectivity in Schizophrenia: A Transdiagnostic Multimodal Meta-analysis of Resting-State Functional and Structural Magnetic Resonance Imaging Studies. Biol Psychiatry. 2019;85(7):573-83. doi:10.1016/j.biopsych.2018.12.003

15. Menon V, Uddin LQ. Saliency, switching, attention and control: a network model of insula function. Brain Struct Funct. 2010;214(5-6):655-67. doi:10.1007/s00429-010-0262-0

16. Uddin LQ. Salience processing and insular cortical function and dysfunction. Nat Rev Neurosci. 2015;16(1):55-61. doi:10.1038/nrn3857

17. Tombaugh TN. Trail Making Test A and B: normative data stratified by age and education. Arch Clin Neuropsychol. 2004;19(2):203-14. doi:10.1016/S0887-6177(03)00039-8

18. Narr KL, Bilder RM, Woods RP, Thompson PM, Szeszko P, Robinson D, et al. Regional specificity of cerebrospinal fluid abnormalities in first episode schizophrenia. Psychiatry Res. 2006;146(1):21-33. doi:10.1016/j.pscychresns.2005.10.005

19. Horga G, Bernacer J, Dusi N, Entis J, Chu K, Hazlett EA, et al. Correlations between ventricular enlargement and gray and white matter volumes of cortex, thalamus, striatum, and internal capsule in schizophrenia. Eur Arch Psychiatry Clin Neurosci. 2011;261(7):467-76. doi:10.1007/s00406-011-0202-x

20. Torres US, Duran FL, Schaufelberger MS, Crippa JA, Louza MR, Sallet PC, et al. Patterns of regional gray matter loss at different stages of schizophrenia: A multisite, cross-sectional VBM study in first-episode and chronic illness. Neuroimage Clin. 2016;12:1-15. doi:10.1016/j.nicl.2016.06.002

21. Honea R, Crow TJ, Passingham D, Mackay CE. Regional deficits in brain volume in schizophrenia: a meta-analysis of voxel-based morphometry studies. Am J Psychiatry. 2005;162(12):2233-45. doi:10.1176/appi.ajp.162.12.2233

22. Yuan R, Biswal BB, Zaborszky L. Functional Subdivisions of Magnocellular Cell Groups in Human Basal Forebrain: Test-Retest Resting-State Study at Ultra-high Field, and Meta-analysis. Cereb Cortex. 2019;29(7):2844-58. doi:10.1093/cercor/bhy150

23. Haxby JV, Connolly AC, Guntupalli JS. Decoding neural representational spaces using multivariate pattern analysis. Annu Rev Neurosci. 2014;37:435-56. doi:10.1146/annurev-neuro-062012-170325

24. Grothe MJ, Scheef L, Bauml J, Meng C, Daamen M, Baumann N, et al. Reduced Cholinergic Basal Forebrain Integrity Links Neonatal Complications and Adult Cognitive Deficits After Premature Birth. Biol Psychiatry. 2017;82(2):119-26. doi:10.1016/j.biopsych.2016.12.008
